# Electrochemical oxidative cyclization of *N*-allylamides for the synthesis of CF_3_-containing benzoxazines and oxazolines†

**DOI:** 10.1039/d3ra07282g

**Published:** 2024-01-02

**Authors:** Yutian Li, Li Wang, Shengbin Zhou, Guoxue He, Yu Zhou

**Affiliations:** a School of Pharmaceutical Science and Technology, Hangzhou Institute for Advanced Study, University of Chinese Academy of Sciences Hangzhou 310024 China heguoxue@ucas.ac.cn; b State Key Laboratory of Drug Research, Shanghai Institute of Materia Medica, Chinese Academy of Sciences Shanghai 201203 China zhouyu@simm.ac.cn; c University of Chinese Academy of Sciences Beijing 100049 China

## Abstract

The introduction of trifluoromethyl (–CF_3_) groups into compounds is a common synthetic strategy in organic chemistry. Commonly used methods for introducing trifluoromethyl groups are limited by harsh reaction conditions, low regioselectivity, or the need for excess reagents. In this study, a facile electrochemical oxidative and radical cascade cyclization of *N*-(2-vinylphenyl)amides for the synthesis of CF_3_-containing benzoxazines and oxazolines was obtained. This sustainable protocol features inexpensive and durable electrodes, a wide range of substrates, diverse functional group compatibility under transition-metal-free, external-oxidant-free, and additive-free conditions, and can be applied in an open environment.

## Abstract 2

A facile electrochemical oxidative and radical cascade cyclization of *N*-(2-vinylphenyl)amides with CF_3_SO_2_Na was conducted to synthesize the CF_3_-containing benzoxazines and oxazolines.

## Graphical abstract

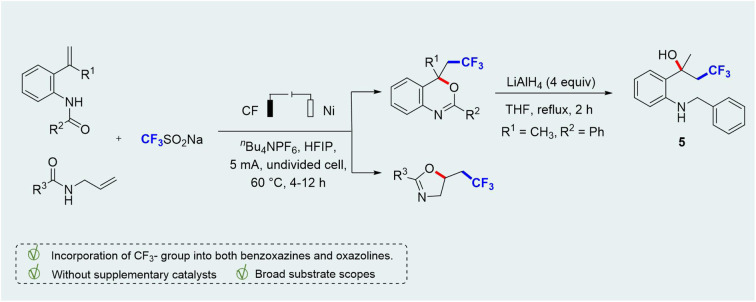

## Introduction

**Fig. 1:**
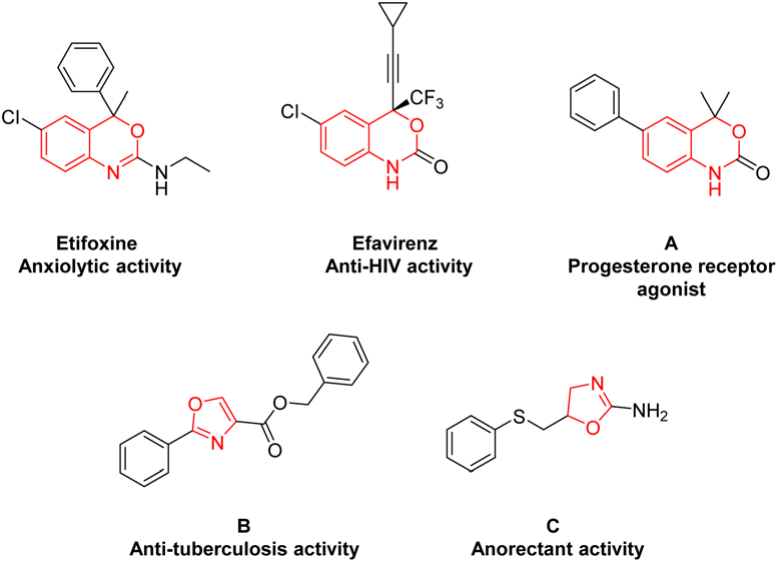
Bioactive compounds containing benzoxazine or oxazoline motifs.

Heterocyclic compounds are one of the most important skeletons in organic synthesis, pharmaceutical chemistry, materials science and bioscience. Heterocycles containing N and O atoms play a crucial role in pharmaceuticals and functional molecules.^1^ Among these, benzoxazines and oxazolines are common privileged fragments frequently found in pharmaceutical molecules and biologically active compounds with remarkable biological activities,^2^ such as anxiolytic,^3^ anti-HIV,^4^ progesterone receptor agonist,^5^ anti-tuberculosis,^6^ and anorectant activities^7^(Fig. 1).

**Scheme 1:**
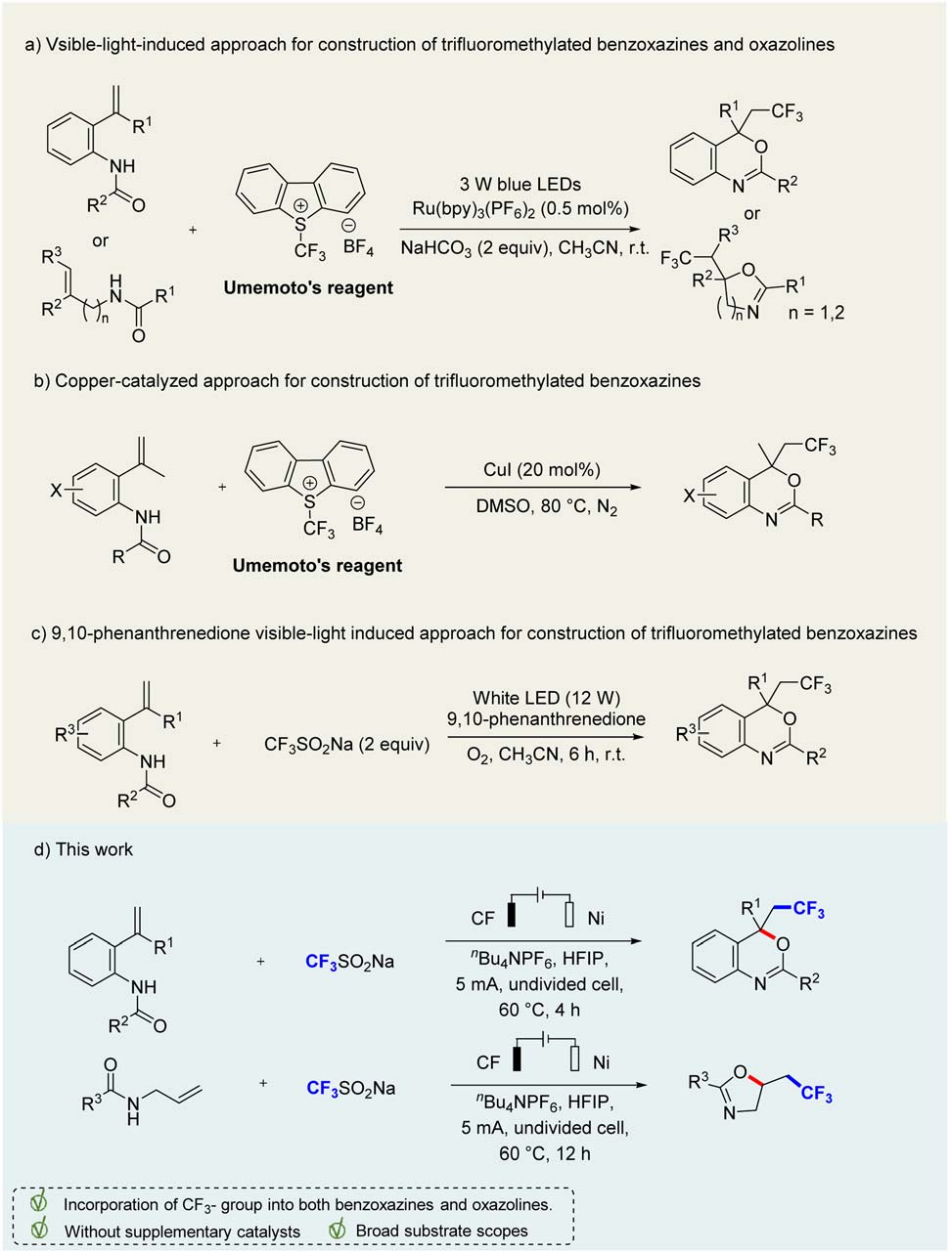
Strategies for the synthesis of trifluoromethylated benzoxazines.

Generally, the incorporation of fluorinated moieties into molecules can significantly change their physical, chemical, and biological properties. For example, the trifluoromethyl (–CF_3_) moiety is widely present in a variety of drugs (celecoxib, fluoxetine, and trifloxystrobin *etc.*), which can improve the efficacy, solubility, lipophilicity, metabolic stability, and binding selectivity.^8^ Efavirenz containing trifluoromethyl benzoxazine shows potent anti-HIV activity.^4^ As a result, the potential values of these trifluoromethylated benzoxazines and oxazolines have attracted significant attention from chemists to develop efficient strategies for the construction of these intriguing molecule scaffolds.^9^ Xiao and co-workers reported a visible-light-induced photocatalytic trifluoromethylation of *N*-allylamides for the synthesis of CF_3_-containing benzoxazines and oxazolines under Umemoto's reagent and Ru(bpy)_3_(PF_6_)_2_ (Scheme 1a).^9*m*^ Similarly, Kumar's group developed a copper-catalyzed approach for construction of trifluoromethylated benzoxazines by using Umemoto's reagent (Scheme 1b).^10^ These methods are effective and versatile, but are limited to transition-metal catalysts and Umemoto's reagent as CF_3_ sources. In addition, Natarajan and colleagues disclosed a novel 9,10-phenanthrenedione visible-light photocatalysis protocol for the synthesis of trifluoromethylated benzoxazines by using *N*-(2-vinylphenyl)amides and trifluoromethylsulfinate under oxygen atmosphere (Scheme 1c).^9*a*^ Nevertheless, it still requires additional photocatalysts and oxidants. Therefore, it is highly desirable to develop alternatively efficient, sustainable, green, and environmentally friendly synthetic methods avoiding transition metal catalysts and chemical oxidants.

Organic electrochemistry provides an effective and sustainable strategy for the synthesis of valuable chemicals, employing inexpensive and renewable electrons as redox reagents.^11^ In our continuous efforts, our goal is to develop green, metal-free, and efficient methods to construct diversified heterocyclic scaffolds.^12^ In our previous work, we reported a direct azidation of benzylic C(sp^3^)–H bonds through an electrochemical process.^13^ Herein, we'd like to report a new finding to construct trifluorinated benzoxazines and oxazolines through an effective electrochemical strategy, which may use cheap carbon fibre and nickel plates as electrodes in an undivided cell, without any external chemical oxidants, metal catalysts and additives (Scheme 1d). However, while we were preparing this manuscript, a similar work appeared, focusing on the construction of CF_2_-substituted benzoxazines,^14^ in which the reaction system required trifluoroacetic acid as a catalyst, adding complexity to the reaction system. In contrast, our reaction system is simpler and environmentally benign without the need for a transition metal catalyst or external oxidant, and can proceed smoothly with diverse functional group compatibility.

## Results and discussion

Based on the above conception, we have attempted to achieve the CF_3_-containing benzoxazines by treatment of *N*-(2-(prop-1-en-2-yl)phenyl)benzamide (1a) with CF_3_SO_2_Na. The reaction was carried out in an undivided cell equipped with a carbon fibre (CF) anode and a nickel plate (Ni) cathode under a constant current of 5 mA (Table 1). The desired product 2a was obtained in 72% yield when ^*n*^Bu_4_NPF_6_ was used as the electrolyte in HFIP at 60 °C for 4 h (entry 1). We tried other electrolytes, such as Et_4_NBF_4_, ^*n*^Bu_4_NOAc, ^*n*^Bu_4_NBr, and ^*n*^Bu_4_NI. Et_4_NBF_4_ resulted in a significant decrease (entry 2) in yield, only trace of the product was observed when using ^*n*^Bu_4_NOAc and ^*n*^Bu_4_NBr as electrolytes (entry 3), and the product was I-containing benzoxazine derivative when using ^*n*^Bu_4_NI as the electrolyte (entry 4). Besides, the product 2a also was observed in the absence of electrolyte (entry 5). When we replaced solvent with DMSO (entry 6), CH_3_CN (entry 7), CH_3_OH (entry 8), and DCE (entry 9), all of them resulted in a slight decrease in the yield. This could be attributed to the ability of HFIP to stabilize radical cation intermediates, thereby aiding in substrate oxidation while preventing the product of overoxidation.^15^ We further evaluated other electrode materials, including Pt plate (entry 10), Fe plate (entry 11) as anode, and Fe plate (entry 12), Al plate (entry 13) as cathode, none of them was more effective. We transformed the current to 2 mA (entry 14) or 10 mA current (entry 15), the reaction efficiency was noticeably dropped in 2 mA current. The product 2a was decreased under 10 mA current, which speculated that high current may cause peroxidation. The reaction temperature also was investigated, which led to lower yields (entries 16–18). When the equivalent of CF_3_SO_2_Na was reduced to 1, it resulted in a slight decrease in the yield (entry 19). Furthermore, electricity (entry 20) was essential for the process of the reaction.

**Table 1:** Optimization of reaction conditions^a^

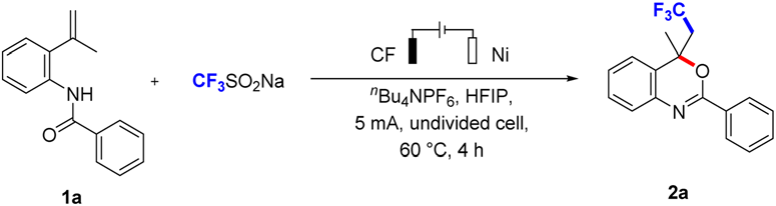		
Entry	Variation from standard conditions	Yield^b^ [%]
**1**	**None**	**72**
2	Et_4_NBF_4_ as electrolyte	39
3	^*n*^Bu_4_NBr or ^*n*^Bu_4_NOAc as electrolyte	Trace
4	^*n*^Bu_4_NI as electrolyte	0
5	No electrolyte	13
6	DMSO as solvent	51
7	CH_3_CN as solvent	38
8	CH_3_OH as solvent	37
9	DCE as solvent	66
10	Pt plate as anode	25
11	Fe plate as anode	17
12	Fe plate as cathode	29
13	Al plate as cathode	56
14	2 mA	42
15	10 mA	60
16	r.t.	35
17	40 °C	64
18	80 °C	59
19	CF_3_SO_2_Na (1 equiv.)	63
20	No electricity	0

a Reaction conditions: undivided cell, 1a (0.25 mmol), CF_3_SO_2_Na (0.5 mmol), solvent (6 mL), ^*n*^Bu_4_NPF_6_ (0.5 mmol), 5 mA, 60 °C, 4 h (3.0 F mol^−1^).

b Isolated yield. Under air atmosphere. CF = Carbon fibre (1 × 1 × 0.01 cm), Pt = platinum (1 × 1 × 0.01 cm), Ni = nickel (1 × 1 × 0.01 cm). HFIP, 1,1,1,3,3,3-hexafluoro-2-propanol, DCE, 1,2-dichloroethane.

**Scheme 2:**
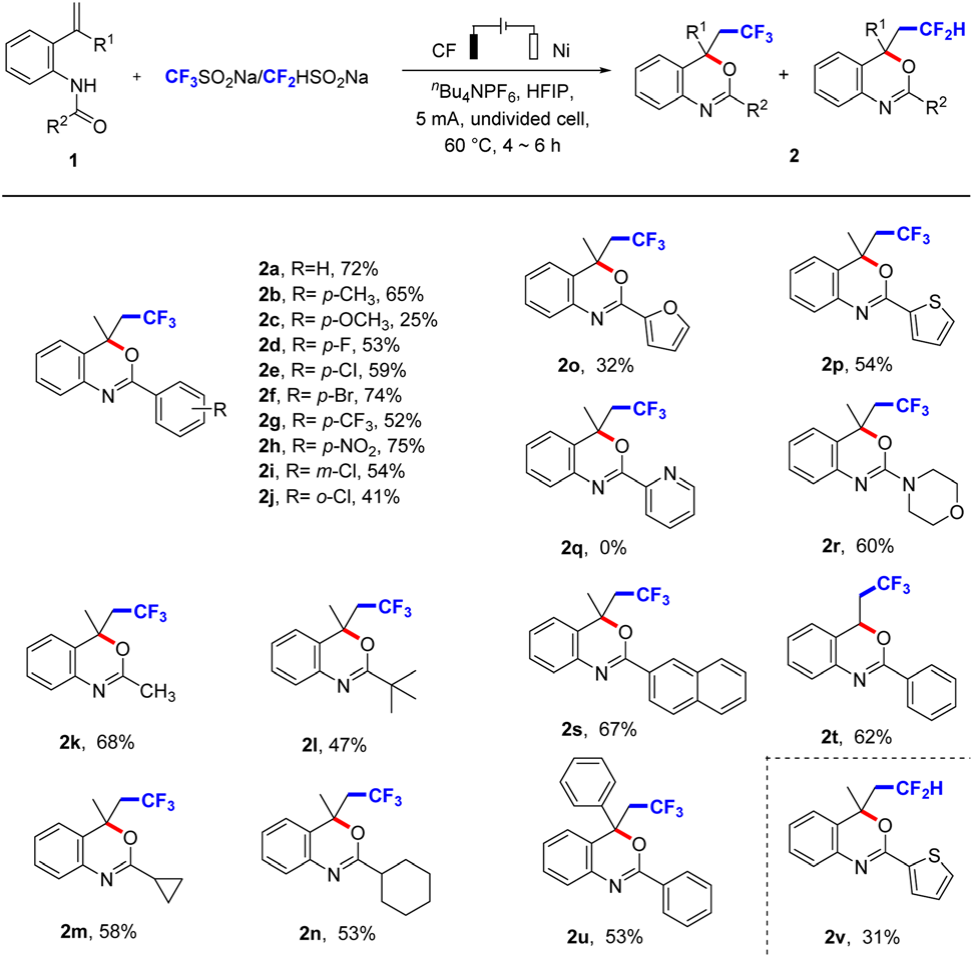
Substrate scope of CF_3_-containing benzoxazines. ^a^Reaction conditions: undivided cell, 1 (0.25 mmol), CF_3_SO_2_Na/CF_2_HSO_2_Na (0.5 mmol), ^*n*^Bu_4_NPF_6_ (0.5 mmol), HFIP (6 mL), under air atmosphere.

With the optimal conditions in hand, the substrate scope of CF_3_-containing benzoxazines was explored (Scheme 2). Firstly, we introduced electron-donating groups or electron-withdrawing groups into *N*-(2-(prop-1-en-2-yl)phenyl)benzamide (1a) and they reacted smoothly to obtain corresponding products 2 in moderate to good yields, such as methyl (2b), methoxy (2c), and halides (2d, 2e, 2f, 2i, and 2j), especially the strong electron-withdrawing groups trifluoromethyl (2g) and nitryl (2h) were all tolerant. Besides, we replaced the R^2^ group by methyl (2k), tertiary butyl (2l), cyclopropyl (2m), cyclohexyl (2n), which reacted smoothly to afford the target product in good yields. Furyl (2o) or thienyl (2p) was transformed into the desired product in moderate yields, but pyridyl (2q) could not produce the target product. We speculated that the electron-withdrawing effect of pyridine made it difficult for 1q to generate the corresponding intermediate I or II. We also introduced morpholinyl (2r) and naphthyl (2s) into R^2^ group, the target products were obtained. Further explorations about the R^1^ group were hydrogen (2t) and phenyl (2u), the corresponding target compounds were also generated and showed great compatibility. In this synthetic system, CF_2_-substituted benzoxazines were also successfully synthesized using CF_2_HSO_2_Na as the difluoromethylation reagent (31% yield for compound 2v), which indicates that the reaction system has good applicability.

**Scheme 3:**
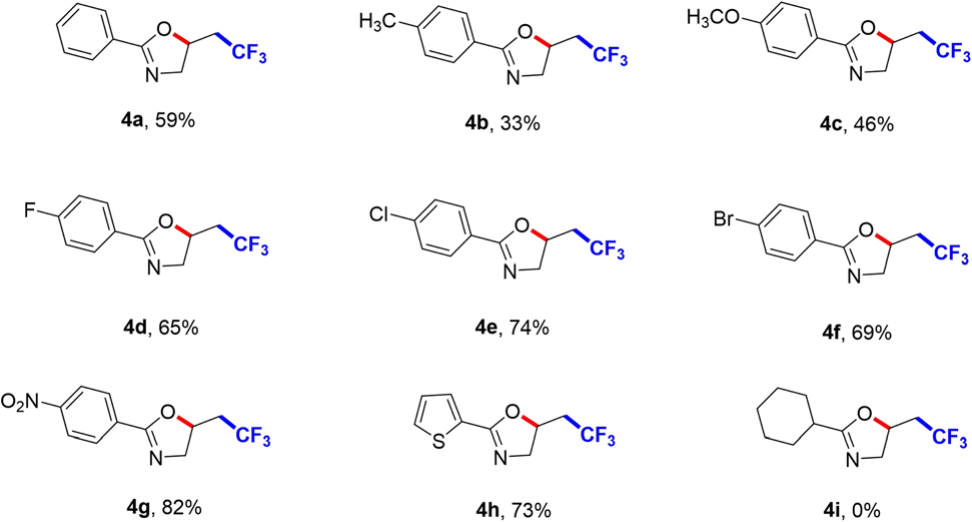
Substrate scope of CF_3_-containing oxazolines. ^a^Reaction conditions: undivided cell, 3 (0.25 mmol), CF_3_SO_2_Na (0.5 mmol), ^*n*^Bu_4_NPF_6_ (0.5 mmol), HFIP (6 mL), under air atmosphere.

We further explored the substrate scope of CF_3_-containing oxazolines, and the results were shown in Scheme 3. *N*-allylbenzamide 3a was reacted with CF_3_SO_2_Na to access the trifluoromethylation product 2-phenyl-5-(2,2,2-trifluoroethyl)-4,5-dihydrooxazole (4a) in 59% yield. *N*-allylbenzamides with various substituents such as methyl (4b), methoxy (4c), halides (4d, 4e and 4f) were all tolerant. The benzene rings with electron-deficient nitryl (4g) gave 82% yield. Meanwhile, when introducing furyl (4h) into the R^3^ group, the corresponding target product also was obtained. But introducing cyclohexyl (4i) into R^3^ group could not produce the target product, which indicated it had a very great influence on this transformation.

**Scheme 4:**
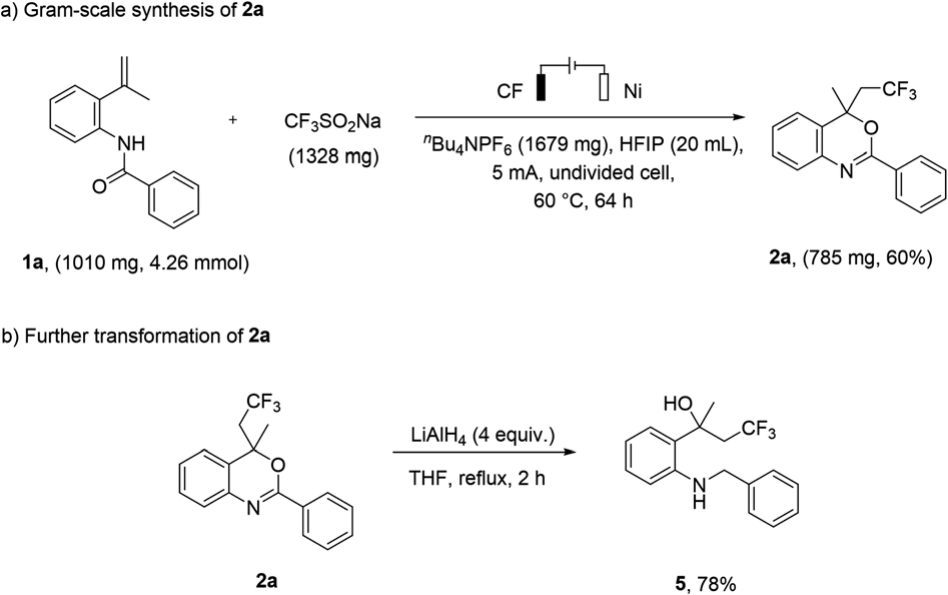
Gram-scale synthesis and further transformation.

To further evaluate the practicality and potential applications of this method, we performed the reaction on a gram-scale preparation with 1a, and the yield of product 2a was 60% under a constant current of 5 mA for 64 h (Scheme 4a). In addition, the product 2a can be further converted into 2-(2-(benzylamino)phenyl)-4,4,4-trifluorobutan-2-ol (5) at a yield of 78% (Scheme 4b).

**Scheme 5:**
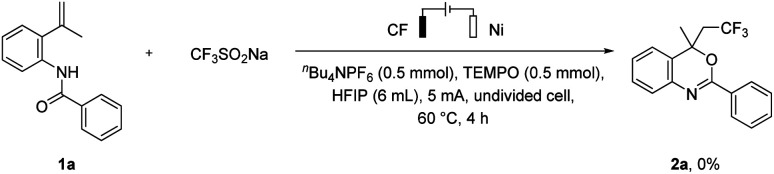
Control experiments of the reaction.

In order to investigate the possible mechanism of this transition, several control experiments were performed. No desired product was obtained when 2,2,6,6-Tetramethylpiperidoxyl (TEMPO) was added (Scheme 5).

**Scheme 6:**
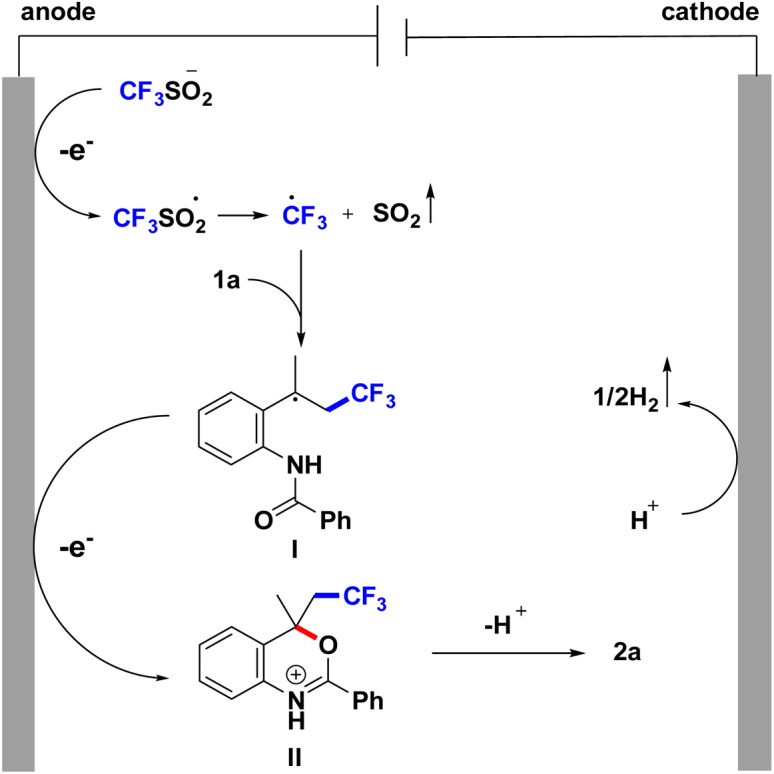
Proposal mechanism of the reaction.

A plausible mechanism for the formation of product has been proposed based on the related reports.^16^ As explained in Scheme 6, initially the HFIP undergoes cathodic reduction to generate hydrogen gas at the cathode. At the anode, CF_3_SO_2_Na produces the CF_3_SO_2_ radical under anodic oxidation and further forms the CF_3_ radical. Subsequently, CF_3_ radicals are added to the double bonds of the olefins to generate the alkyl radical intermediate I. I undergoes a radical cyclization and anodic oxidation to furnish intermediate II. Afterwards, the intermediate II is finally converted into CF_3_-containing benzoxazine 2a by deprotonation (Scheme 6).

**Fig. 2:**
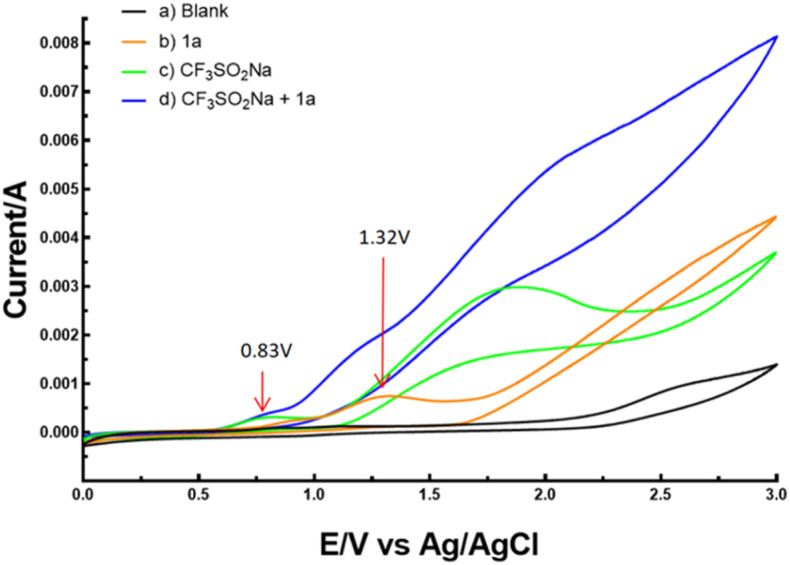
Cyclic voltammetric experiments of 1a and CF_3_SO_2_Na.

To justify the proposed reaction pathway outlined in Scheme 6, we conducted cyclic voltammetric (CV) experiments. As shown in Fig. 2, the oxidation peak of CF_3_SO_2_Na was 0.83 V, and 1a had an oxidation peak of 1.32 V. These results indicated that CF_3_SO_2_Na was oxidized preferentially at the anode (see the ESI† for details).

## Conclusions

In summary, we have developed a mild and efficient electrochemical oxidative and radical cascade cyclization of olefinic amides to afford trifluorinated benzoxazines and oxazolines using cheap and durable nickel plates as electrodes. This paper presents a simple, practical, green and environmentally benign protocol for the synthesis of fluorinated benzoxazines and oxazolines. In the absence of any transition metal catalysts, external oxidizers and additives, this protocol proceeds smoothly with diverse functional group compatibility.

## Conflicts of interest

There are no conflicts to declare.

## Supplementary Material

RA-014-D3RA07282G-s001

## References

[cit1] Eichenbaum G., Zhou J., Kelley M. F., Roosen W., Costa-Giomi P., Louden C., Di Prospero N. A., Pandina G., Singh J. B., Ford L., Moyer J. A., Nork T. M., Ver Hoeve J. N., Aguirre G. D. (2014). Implications of retinal effects observed in chronic toxicity studies on the clinical development of a CNS-active drug candidate. Regul. Toxicol. Pharmacol..

[cit2] Ilaš J., Jakopin Ž., Borštnar T., Stegnar M., Kikelj D. (2008). 3,4-Dihydro-2H-1,4-benzoxazine Derivatives Combining Thrombin Inhibitory and Glycoprotein IIb/IIIa Receptor Antagonistic Activity as a Novel Class of Antithrombotic Compounds with Dual Function. J. Med. Chem..

[cit3] Schlichter R., Rybalchenko V., Poisbeau P., Verleye M., Gillardin J.-M. (2000). Modulation of GABAergic synaptic transmission by the non-benzodiazepine anxiolytic etifoxine. Neuropharmacology.

[cit4] Bastos M. M., Costa C. C. P., Bezerra T. C., da Silva F. d. C., Boechat N. (2016). Efavirenz a nonnucleoside reverse transcriptase inhibitor of first-generation: Approaches based on its medicinal chemistry. Eur. J. Med. Chem..

[cit5] Zhang P., Terefenko E. A., Fensome A., Wrobel J., Winneker R., Lundeen S., Marschke K. B., Zhang Z. (2002). 6-Aryl-1,4-dihydro-benzo[*d*][1,3]oxazin- 2-ones: A Novel Class of Potent, Selective, and Orally Active Nonsteroidal Progesterone Receptor Antagonists. J. Med. Chem..

[cit6] Jin Z. (2009). Muscarine, imidazole, oxazole and thiazole alkaloids. Nat. Prod. Rep..

[cit7] Freiter E. R., Abdallah A. H., Strycker S. J. (1973). 2-Amino-5-substituted oxazolines and intermediates as potential anorectants. J. Med. Chem..

[cit8] Müller K., Faeh C., Diederich F. (2007). Fluorine in Pharmaceuticals: Looking Beyond Intuition. Science.

[cit9] Natarajan P., Chuskit D., Priya, Manjeet (2022). Transition-metal-free synthesis of trifluoromethylated benzoxazines *via* a visible-light-promoted tandem difunctionalization of o-vinylanilides with trifluoromethylsulfinate. New J. Chem..

[cit10] Jana S., Ashokan A., Kumar S., Verma A., Kumar S. (2015). Copper-catalyzed trifluoromethylation of alkenes: synthesis of trifluoromethylated benzoxazines. Org. Biomol. Chem..

[cit11] Zhu C., Ang N. W. J., Meyer T. H., Qiu Y., Ackermann L. (2021). Organic Electrochemistry: Molecular Syntheses with Potential. ACS Cent. Sci..

[cit12] He G., Ma J., Zhou J., Li C., Liu H., Zhou Y. (2021). A metal-free method for the facile synthesis of indanones *via* the intramolecular hydroacylation of 2-vinylbenzaldehyde. Green Chem..

[cit13] He G., Li Y., Zhou S., Yang X., Shang A., Wang Y., Liu H., Zhou Y. (2022). A Facile Electrochemical Strategy for the Azidation of Benzylic C(sp3)–H Bonds. Eur. J. Org Chem..

[cit14] Chen X., Jiang J., Huang X.-J., He W.-M. (2023). Electrochemical oxidative radical cascade reactions for the synthesis of difluoromethylated benzoxazines. Org. Chem. Front..

[cit15] Eberson L., Hartshorn M. P., Radner F., Persson O. (1996). Persistent radical cation solutions from the reaction between aromatics and bromine, chlorine or iodine chloride in 1,1,1,3,3,3-hexafluoropropan-2-ol at room temperature. Chem. Commun..

[cit16] Zhang S., Li L., Zhang J., Zhang J., Xue M., Xu K. (2019). Electrochemical fluoromethylation triggered lactonizations of alkenes under semi-aqueous conditions. Chem. Sci..

