# Supplementary material for: Electrochemical oxidative cyclization of N-allylamides for the synthesis of CF3-containing benzoxazines and oxazolines
Source: RSC Adv. 2024 Jan 2;14(1):154–9. doi: 10.1039/d3ra07282g (PMC10758801; doi:10.1039/d3ra07282g)
Supplement: RA-014-D3RA07282G-s001 [file RA-014-D3RA07282G-s001.pdf]

Electronic Supplementary Material (ESI) for RSC Advances.
This journal is © The Royal Society of Chemistry 2023

Electrochemical Oxidative Cyclization of *N*-allylamides for the Synthesis of CF_3-Containing Benzoxazines and Oxazolines

Yutian Li,[a,c,] Li Wang,[a,c] Shengbin Zhou,[a,c] Guoxue He,[a,c,]* and Yu Zhou[a,b,c,]*

[a]School of Pharmaceutical Science and Technology, Hangzhou Institute for Advanced Study, UCAS, Hangzhou 310024, China.

[b]State Key Laboratory of Drug Research, Shanghai Institute of Materia Medica, Chinese Academy of Sciences, Shanghai 201203, China.

[c]University of Chinese Academy of Sciences, Beijing 100049, China.

E-mail: heguoxue@ucas.ac.cn; zhouyu@simm.ac.cn.

Content

1. General Experimental Information S2-S2
2. General Procedures for the Reactions S2-S5
3. Characterization Data for the Products S5-S14
4. ^{1}H NMR, ^{13}C NMR and ^{19}F NMR Spectra S15-S35
5. Cyclic Voltammogram S36-S36
6. References S37-S37

1. General Experimental Information

Benzamides derivatives (**1**) and (**3**) were synthesized in round bottom flasks with a stirring bar and were purified by flash chromatography. Unless otherwise specified, the raw materials were purchased from commercial sources, and used without further purification. TLC was performed on silica gel GF_{254} plates (Qingdao Marine Chemical Co., Ltd., China) and was visualized with UV lamp (254 and 365 nm). Flash column chromatography was performed on silica gel (300 - 400 mesh). **^{1}H NMR** spectra were recorded at 400 and 500 MHz NMR spectrometer (Bruker). **^{13}C NMR** spectra were recorded at 100 and 125 MHz NMR spectrometer. **^{19}F NMR** spectra were recorded at 376 and 470 MHz NMR spectrometer. The ^{1}H, ^{13}C and ^{19}F chemical shifts are referenced to signals at δ 0.00 (TMS). ^{1}H NMR coupling constants (*J*) are reported in Hertz (Hz) and multiplicities are indicated as follows: s (singlet), d (doublet), t (triplet) and m (multiple). High resolution mass spectra (HRMS, ESI) were measured on a Micromass Ultra Q-TOF spectrometer. All new products were identified through **^{1}H NMR**, **^{13}C NMR**, **^{19}F NMR** and **HRMS**.

2. General Procedures for the Reactions

General Procedures for Preparation of Compounds 1a-1s

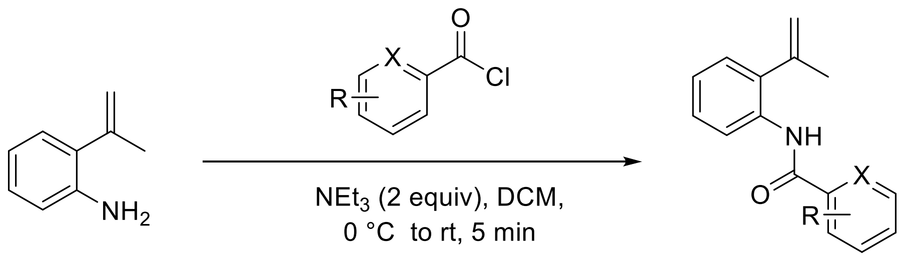

1a-1s

Taking *N*-(2-(prop-1-en-2-yl)phenyl)benzamide (**1a**) for an example, to a stirred solution of 2-isopropenylaniline (0.848 g, 7.5 mmol) and triethylamine (2.0 mL, 15 mmol) in dry CH_2Cl_2 (25 mL), benzoyl chloride (2.108 g, 15 mmol) was added dropwise using a dropping funnel at 0 ℃. The reaction mixture was stirred at room temperature for 5 min. After completion, the reaction was washed by 10 mol % aqueous HCl solution (15 mL), saturated aqueous $NaHCO_3$ solution (15 mL), brine (25 mL) and dried over Na_2SO_4. The organic solvent was removed by rotary evaporator under vacuum and the residue was purified by column chromatography on silica gel using PE/EA (10:1). A white solid was obtained (1.337 g, yield: 75%).

General Procedures for Preparation of Compounds 1t-1u

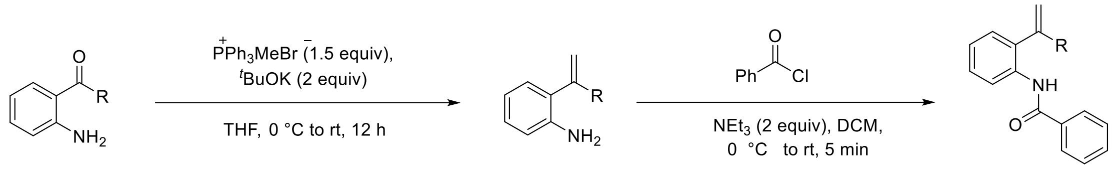

1t-1u

Taking *N*-(2-vinylphenyl)benzamide (**1t**) for an example, to a stirred solution of PPh_3MeBr (8.573 g, 24 mmol) in dry THF (15 mL), ^tBuOK (3.591 g, 32 mmol) was added in portions under nitrogen. After the mixture was stirred at room temperature for 1 h, a solution of 2-aminobenzaldehyde (1.938g, 16 mmol) in THF (15 mL) was added dropwise. The reaction mixture was then stirred overnight at room temperature under nitrogen. The reaction mixture was quenched with water and extracted with EtOAc (100 mL). The combined organic layers were washed with saturated $NaHCO_3$ (50 mL) and brine (50 mL), dried over anhydrous Na_2SO_4, and concentrated on rotary evaporator under vacuum and the residue was purified by column chromatography on silica gel using PE/EA (16:1). A white solid was obtained (1.220 g, yield: 64%).

To a stirred solution of 2-vinylaniline (1.220 g, 10 mmol) and triethylamine (3.0 mL, 20 mmol) in dry CH_2Cl_2 (25 mL), benzoyl chloride (2.811 g, 20 mmol) was added dropwise using a dropping funnel at 0 °C. The reaction mixture was stirred at room temperature for 15 min. After completion, the reaction was washed by 10 mol % aqueous HCl solution (20 mL), saturated aqueous $NaHCO_3$ solution (20 mL), brine (30 mL) and dried over Na_2SO_4. The organic solvent was removed by rotary evaporator under vacuum and the residue was purified by column chromatography on silica gel using PE/EA (10:1). A white solid was obtained (1.800 g, yield: 82%).

General Procedures for Preparation of *N*-allylbenzamides (3a-3i)

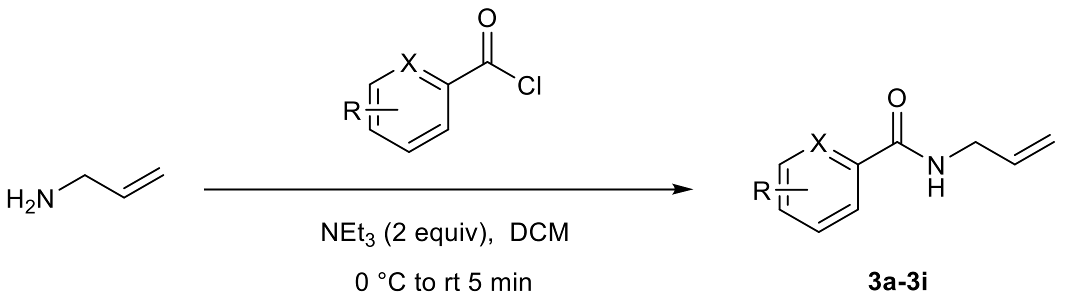

3a-3i

Taking *N*-allylbenzamide (**3a**) for an example, to a stirred solution of prop-2-en-1-amine (0.428 g, 7.5 mmol) and triethylamine (2.0 mL, 15 mmol) in dry CH_2Cl_2 (25 mL), benzoyl chloride (2.108 g, 15 mmol) was added dropwise using a dropping funnel at 0 °C. The reaction mixture was stirred at room temperature for 5

min. After completion, the reaction was washed by 10 mol % aqueous HCl solution (15 mL), saturated aqueous $NaHCO_3$ solution (15 mL), brine (25 mL) and dried over Na_2SO_4. The organic solvent was removed by rotary evaporator under vacuum and the residue was purified by column chromatography on silica gel using PE/EA (6:1). A white solid was obtained (0.803 g, yield: 66%).

General Procedures for the Synthesis of Target Products (2a as an example)

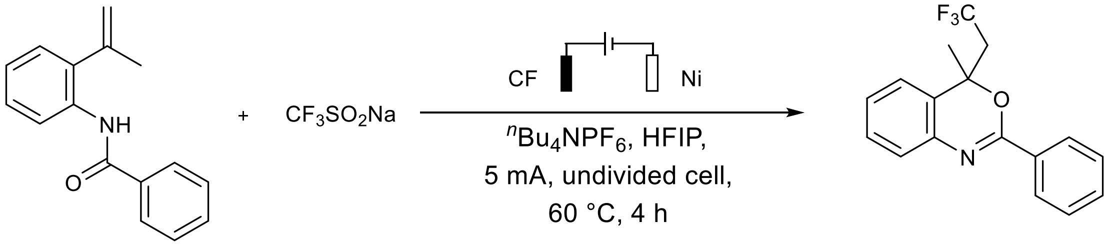

Taking 4-methyl-2-phenyl-4-(2, 2, 2-trifluoromethyl)[d][1,3]oxazine (**2a)** for an example, in a two-necked round-bottomed flask (25 mL) equipped with a stirring bar (Figure S1), *N*-acyl-(2-ene)-aniline **1a** (0.059 g, 0.25 mmol), CF_3SO_2Na (0.078 g, 0.5 mmol), $^nBu_4NPF_6$ (0.194 g, 0.5 mmol), HFIP (6 mL) were added. The flask was equipped with a carbon fibre (CF) anode (10 mm × 10 mm × 0.1 mm) and a nickel plate (Ni) cathode (10 mm × 10 mm × 0.1 mm), and the distance between the two electrodes was 0.5 cm. The reaction mixture was stirred at a constant current of 5 mA under 60 ℃ for 4 h. After completion, the solvent was concentrated under reduced pressure, and the corresponding products were purified by flash chromatography on silica gel (0.055 g, 72%).

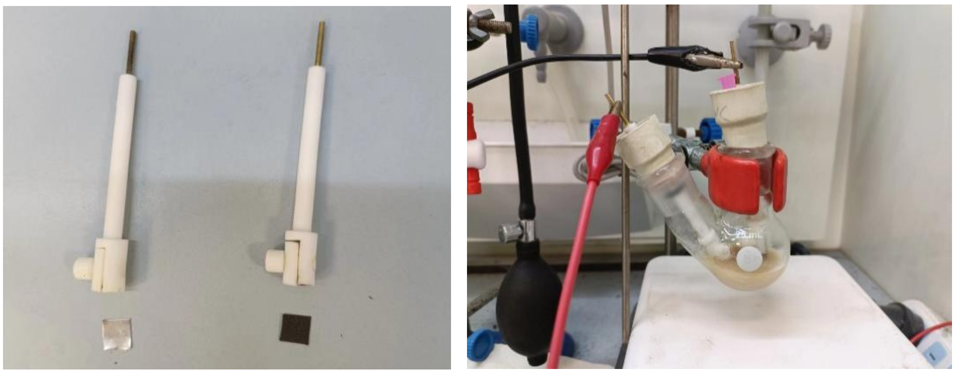

Figure S1. Electrolysis cell for small scale reactions.

General Procedures for the Gram-scale Synthesis

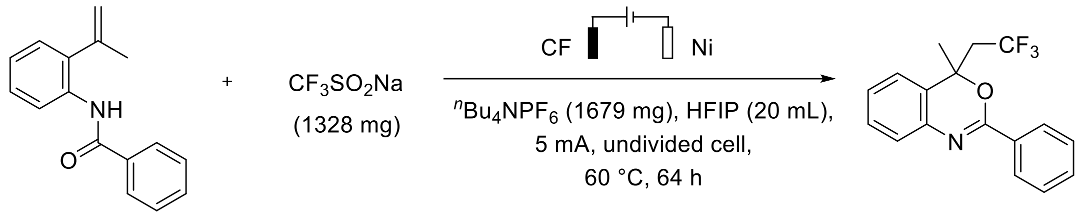

1a, (1010 mg, 4.26mmol) **2a**, (785 mg, 61%)

In a two-necked round-bottomed flask (50 mL) equipped with a stirring bar, *N*-acyl-(2-ene)-aniline **1a** (1.010 g, 4.26 mmol), CF_3SO_2Na (1.328 g, 4.33 mmol), $^nBu_4NPF_6$ (1.679 g, 0.5 mmol), HFIP (20 mL) were added. The flask was equipped with a carbon fibre (CF) anode (20 mm × 20 mm × 0.1 mm) and a nickel plate (Ni) cathode (20 mm × 20 mm × 0.1 mm), and the distance between the two electrodes was 1.0 cm. The reaction mixture was stirred and electrolyzed at a constant current of 5 mA under 60 ℃ for 64 h. After completion, the solvent was concentrated under reduced pressure, and the corresponding products were purified by flash chromatography on silica gel (0.785 g, 60%).

General Procedures for the Further Transformation

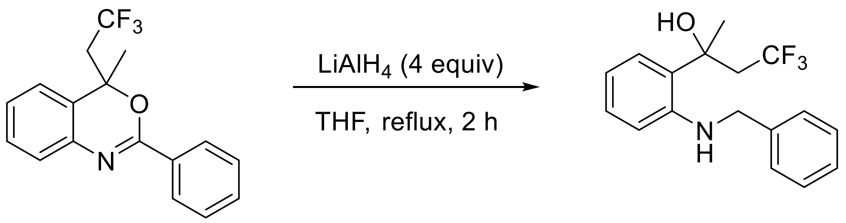

2a **5**, 78%

In a round-bottomed flask (100 mL) equipped with a stirring bar, anhydrous THF (20 mL) was added, $LiAlH_4$ (0.200 g, 5.25 mmol) was added in batches under 0 ℃. Then a solution of **2a** (0.400 g, 1.31 mmol) in anhydrous THF was added slowly. The mixture was refluxed for 2 h. The reaction was quenched with water under an ice bath. The reaction solution was transferred to a separator funnel and 15 mL of 30% NaOH and $MgSO_4$ were added successively.Filter to collect the organic phase, and then concentrate the filtrate under vacuum to obtain 2-(2-(benzylamino)phenyl)-4,4,4-trifluorobutan-2-ol **5** (0.316 g, yield 78%) .

3. Characterization Data for the Products

The products **2a**[1], **2b**[1], **2c**[1], **2d**[1], **2e**[1], **2f**[1], **2h**[1], **2i**[1], **2j**[1], **2k**[1], **2l**[1], **2o**[1], **2p**[1], **2r**[2], **2s**[1], **2t**[1], **2u**[1], **2v**[4], **4a**[3], **4b**[3], **4c**[3], **4d**[3], **4e**[3], and **4f**[3]are known.

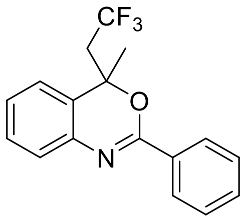

4-methyl-2-phenyl-4-(2, 2, 2-trifluoromethyl)[d][1,3]oxazine (2a): White solid, yield (0.055 g, 72%), ^{1}H NMR (500 MHz, $CDCl_3$), δ 8.18 (d, J = 8.4 Hz, 2H), 7.57 - 7.41 (m, 3H), 7.36 (d, J = 4.4 Hz, 2H), 7.25 - 7.20 (m, 1H), 7.14 (d, J = 7.7 Hz, 1H), 2.94 - 2.81 (m, 1H), 2.70 - 2.57 (m, 1H), 1.93 (s, 3H), ^{13}C NMR[1], ^{19}F NMR[1], HRMS (ESI), m/z calcd for $C_{17}H_{14}F_3NO$ $[M+H]^+$: 306.1100, found: 306.1104.

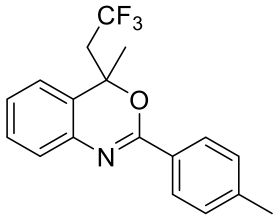

4-methyl-2-(p-tolyl)-4-(2,2,2-trifluoroethyl)-4H-benzo[*d*][1,3]oxazine (2b): White semi-solid, yield (0.052 g, 65%), ^{1}H NMR (500 MHz, $CDCl_3$), δ 8.05 (d, J = 8.0 Hz, 2H), 7.34 - 7.24 (m, 2H), 7.21 (d, J = 8.0 Hz, 2H), 7.18 - 7.13 (m, 1H), 7.07 (d, J = 7.7 Hz, 1H), 2.89 - 2.72 (m, 1H), 2.62 - 2.49 (m, 1H), 2.37 (s, 3H), 1.87 (s, 3H), ^{13}C NMR[1], ^{19}F NMR[1], HRMS (ESI), m/z calcd for $C_{18}H_{16}F_3NO$ $[M+H]^+$: 320.1257, found: 320.1259.

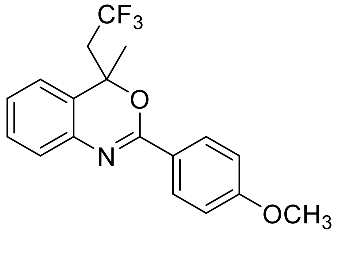

2-(4-methoxyphenyl)-4-methyl-4-(2,2,2-trifluoroethyl)-4H-benzo[d][1,3]oxazine (2c): White semi-solid, yield (0.021 g, 25%), ^{1}H NMR (500 MHz, CD_3OD), δ 8.15 - 7.96 (m, 2H), 7.38 - 7.27 (m, 2H), 7.25 (d, J = 1.3 Hz, 1H), 7.23 (d, 1H), 7.06 - 6.95 (m, 2H), 3.86 (s, 3H), 3.05 - 2.95 (m, 1H), 2.95 - 2.85 (m, 1H), 1.85 (s, 3H), ^{13}C NMR[1], ^{19}F NMR[1], HRMS (ESI), m/z calcd for $C_{18}H_{16}F_3NO_2$ $[M+H]^+$: 336.1206, found: 336.1205.

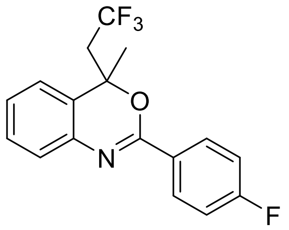

2-(4-fluorophenyl)-4-methyl-4-(2,2,2-trifluoroethyl)-4H-benzo[d][1,3]oxazine (2d): White solid, yield (0.043 g, 53%), ^{1}H NMR (500 MHz, CD_3OD), δ 8.24 - 8.10 (m, 2H), 7.35 - 7.28 (m, 2H), 7.29 - 7.24 (m, 2H), 7.23 - 7.17 (m, 2H), 3.07 - 2.97 (m, 1H), 2.97 - 2.86 (m, 1H), 1.86 (s, 3H), ^{13}C NMR[1], ^{19}F NMR[1], HRMS (ESI), m/z calcd for $C_{17}H_{13}F_4NO$ $[M+H]^+$: 324.1006, found: 324.1005.

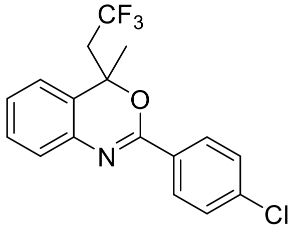

2-(4-chlorophenyl)-4-methyl-4-(2,2,2-trifluoroethyl)-4H-benzo[d][1,3]oxazine (2e): White solid, yield (0.050 g, 59%), ^{1}H NMR (500 MHz, CD_3OD), δ 8.12 - 8.04 (m, 2H), 7.52 - 7.44 (m, 2H), 7.37 - 7.29 (m, 2H), 7.29 - 7.23 (m, 2H), 3.06 - 2.96 (m, 1H), 2.96 - 2.85 (m, 1H), 1.86 (s, 3H), ^{13}C NMR[1], ^{19}F NMR[1], HRMS (ESI), m/z calcd for $C_{17}H_{13}ClF_3NO$ $[M+H]^+$: 340.0711, found: 340.0709.

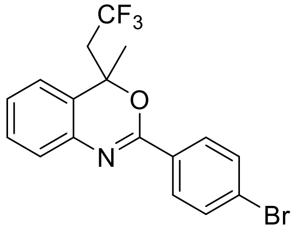

2-(4-bromophenyl)-4-methyl-4-(2,2,2-trifluoroethyl)-4H-benzo[d][1,3]oxazine (2f): Pale yellow semi-solid, yield (0.071 g, 74%), ^{1}H NMR (500 MHz, CD_3OD), δ 8.04 - 7.98 (m, 2H), 7.69 - 7.60 (m, 2H), 7.39 - 7.30 (m, 2H), 7.27 (t, J = 7.4 Hz, 2H), 3.09 - 2.97 (m, 1H), 2.97 - 2.87 (m, 1H), 1.87 (s, 3H), ^{13}C NMR[1], ^{19}F NMR[1], HRMS (ESI), m/z calcd for $C_{17}H_{13}BrF_3NO$ $[M+H]^+$: 384.0206, found: 384.0204.

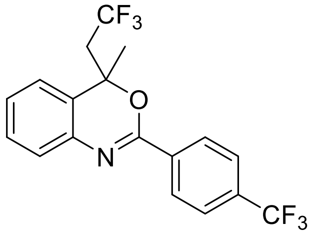

4-methyl-4-(2,2,2-trifluoroethyl)-2-(4-(trifluoromethyl)phenyl)-4H-benzo[d][1,3] oxazine (2g): White solid, yield (0.049 g, 52%), ^{1}H NMR (500 MHz, CD_3OD), δ 8.29 (d, J = 8.2 Hz, 2H), 7.78 (d, J = 8.3 Hz, 2H), 7.40 - 7.31 (m, 2H), 7.31 - 7.25 (m, 2H), 3.09 - 2.99 (m, 1H), 2.99 - 2.88 (m, 1H), 1.88 (s, 3H), ^{13}C NMR (125 MHz, CD_3OD), δ 155.7, 138.7, 137.3, 134.0 (q, $J_{C,F}$ = 32.6 Hz), 130.4, 129.4, 129.3, 128.9, 126.9 (q, J = 275.5 Hz, CF_3), 126.6, 126.3 (q, J = 3.8 Hz, CF_3), 125.4 (q, J = 268.8 Hz, CF_3), 124.7, 77.7 (q, J = 2.4 Hz), 43.2 (q, J = 27.0 Hz, CH_2CF_3), 27.2 (d, J = 1.3 Hz), ^{19}F NMR (470 MHz, CD_3OD), δ -61.5 (t, J = 10.8 Hz, 3F), -65.4, HRMS (ESI), m/z calcd for $C_{18}H_{13}F_6NO$ $[M+H]^+$: 374.0974, found: 374.0973.

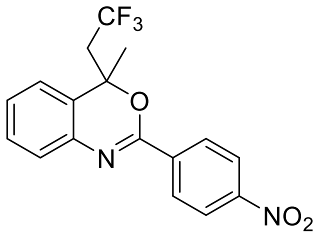

4-methyl-2-(4-nitrophenyl)-4-(2,2,2-trifluoroethyl)-4H-benzo[d][1,3]oxazine (2h): Light yellow-solid, yield (0.066 g, 75%), ^{1}H NMR (500 MHz, CD_3OD), δ 8.33 (s, 4H), 7.39 - 7.33 (m, 2H), 7.33 - 7.28 (m, 2H), 3.11 - 3.00 (m, 1H), 3.00 - 2.89 (m, 1H), 1.89 (s, 3H), ^{13}C NMR[1], ^{19}F NMR[1], HRMS (ESI), m/z calcd for $C_{17}H_{13}F_3N_2O_3$ $[M+H]^+$: 351.0951, found: 351.0954.

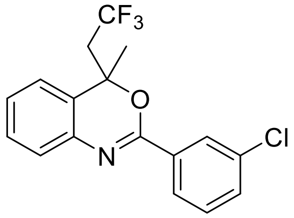

2-(3-chlorophenyl)-4-methyl-4-(2,2,2-trifluoroethyl)-4H-benzo[d][1,3]oxazine (2i): White semi-solid, yield (0.046 g, 54%), ^{1}H NMR (400 MHz, CD_3OD), δ 8.08 (t, J = 1.8 Hz, 1H), 8.04 - 7.98 (m, 1H), 7.59 - 7.50 (m, 1H), 7.49 - 7.41 (m, 1H), 7.39 - 7.22 (m, 4H), 3.10 - 2.97 (m, 1H), 2.97 - 2.84 (m, 1H), 1.86 (s, 3H), ^{13}C NMR[1], ^{19}F NMR[1], HRMS (ESI), m/z calcd for $C_{17}H_{13}ClF_3NO$ $[M+H]^+$: 340.0711, found: 340.0708.

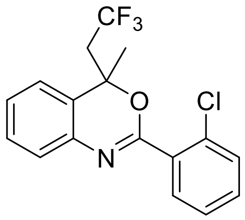

2-(2-chlorophenyl)-4-methyl-4-(2,2,2-trifluoroethyl)-4H-benzo[d][1,3]oxazine (2j): Light brown semi-solid, yield (0.035 g, 41%), ^{1}H NMR (500 MHz, CD_3OD) δ 7.68 (dd, J = 7.7, 1.7 Hz, 1H), 7.56 - 7.45 (m, 2H), 7.41 (td, 1H), 7.38 - 7.26 (m, 3H), 7.21 (dd, J = 7.7, 1.4 Hz, 1H), 3.22 - 3.08 (m, 1H), 3.04 - 2.91 (m, 1H), 1.87 (s, 3H), ^{13}C NMR[1], ^{19}F NMR[1], HRMS (ESI), m/z calcd for $C_{17}H_{13}ClF_3NO$ $[M+H]^+$: 340.0711, found: 340.0714.

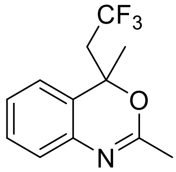

2,4-dimethyl-4-(2,2,2-trifluoroethyl)-4H-benzo[d][1,3]oxazine (2k): Pale yellow semi-solid, yield (0.041 g, 68%), ^{1}H NMR (500 MHz, $CDCl_3$) δ 7.28 (t, J = 7.6 Hz, 1H), 7.23 - 7.11 (m, 2H), 7.05 (d, J = 7.6 Hz, 1H), 2.79 - 2.63 (m, 1H), 2.56 - 2.38 (m, 1H), 2.13 (s, 3H), 1.81 (s, 3H), ^{13}C NMR[1], HRMS (ESI), m/z calcd for $C_{12}H_{12}F_3NO$ $[M+H]^+$: 244.0944, found: 244.0943.

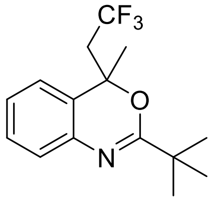

2-(tert-butyl)-4-methyl-4-(2,2,2-trifluoroethyl)-4H-benzo[d][1,3]oxazine (2l): Yellow semi solid, yield (0.034 g, 47%), ^{1}H NMR (500 MHz, CD_3OD) δ 7.27 (td, J = 7.4, 1.8 Hz, 1H), 7.24 - 7.17 (m, 2H), 7.14 (d, J = 7.7 Hz, 1H), 3.11 - 2.95 (m, 1H), 2.95 - 2.81 (m, 1H), 1.67 (s, 3H), 1.26 (s, 9H), ^{13}C NMR[1], ^{19}F NMR[1], HRMS (ESI), m/z calcd for $C_{15}H_{18}F_3NO$ $[M+H]^+$: 286.1413, found: 286.1414.

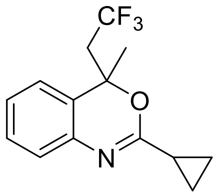

2-cyclopropyl-4-methyl-4-(2,2,2-trifluoroethyl)-4H-benzo[d][1,3]oxazine (2m): White solid, yield (0.039 g, 58%), ^{1}H NMR (400 MHz, CD_3OD) δ 7.31 - 7.24 (m, 1H), 7.24 - 7.14 (m, 2H), 7.06 (dd, J = 7.8, 1.3 Hz, 1H), 3.03 - 2.88 (m, 1H), 2.88 - 2.74 (m, 1H), 1.74 - 1.69 (m, 1H), 1.67 (s, 3H), 1.15 - 0.98 (m, 2H), 0.97 - 0.83 (m, 2H), ^{13}C NMR (100 MHz, CD_3OD), δ 164.4, 138.4, 130.2, 127.6, 126.8 (q, J = 277 Hz, CF_3), 124.7, 124.5, 78.6 (q, J = 2.4 Hz), 44.3 (q, J = 27.0 Hz, CH_2CF_3), 28.8 (d, J = 1.0 Hz),

15.1, 7.1, 7.0, ^{19}F NMR (376 MHz, CD_3OD), δ -61.5 (t, J = 10.7 Hz, 3F), HRMS (ESI), m/z calcd for $C_{14}H_{14}F_3NO$ $[M+H]^+$: 270.1100, found: 270.1104.

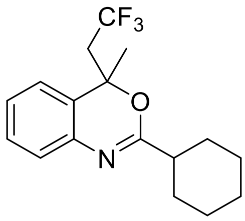

2-cyclohexyl-4-methyl-4-(2,2,2-trifluoroethyl)-4H-benzo[d][1,3]oxazine (2n): White solid, yield (0.041 g,53%), ^{1}H NMR (500 MHz, CD_3OD) δ 7.26 (qd, J = 7.6, 1.9 Hz, 1H), 7.23 - 7.16 (m, 2H), 7.09 (dd, J = 7.8, 1.3 Hz, 1H), 3.06 - 2.93 (m, 1H), 2.91 - 2.77 (m, 1H), 2.30 (tt, J = 11.9, 3.5 Hz, 1H), 1.95 - 1.88 (m, 2H), 1.88 - 1.78 (m, 2H), 1.70 (s, 3H), 1.59 - 1.46 (m, 2H), 1.42 - 1.20 (m, 4H), ^{13}C NMR (125 MHz, CD_3OD), δ 166.7, 138.3, 130.1, 128.4, 127.9, 127.0 (q, J = 278.8 Hz, CF_3), 125.1, 124.8, 78.3 (q, J = 2.5 Hz), 45.3, 44.6 (q, J = 26.9 Hz, CH_2CF_3), 30.5, 30.5, 29.3 (d, J = 1.3 Hz), 27.0, 26.8, 26.8, ^{19}F NMR (470 MHz, CD_3OD), δ -61.4 (t, J = 10.7 Hz, 3F), HRMS (ESI), m/z calcd for $C_{17}H_{20}F_3NO$ $[M+H]^+$: 312.1570, found: 312.1573.

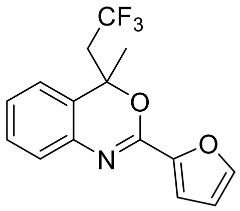

2-(furan-2-yl)-4-methyl-4-(2,2,2-trifluoroethyl)-4H-benzo[d][1,3]oxazine (2o): White solid, yield (0.024 g, 32%), ^{1}H NMR (500 MHz, CD_3OD) δ 7.77 (d, J = 1.8 Hz, 1H), 7.40 - 7.29 (m, 2H), 7.29 - 7.22 (m, 2H), 7.14 (d, J = 3.5 Hz, 1H), 6.63 (dd, J = 3.5, 1.8 Hz, 1H), 3.05 - 2.92 (m, 1H), 2.92 - 2.80 (m, 1H), 1.85 (s, 3H), ^{13}C NMR[1], ^{19}F NMR[1], HRMS (ESI), m/z calcd for $C_{15}H_{12}F_3NO_2$ $[M+H]^+$: 296.0893, found: 296.0896.

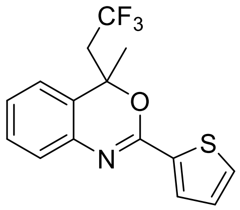

4-methyl-2-(thiophen-2-yl)-4-(2,2,2-trifluoroethyl)-4H-benzo[d][1,3]oxazine (2p): White semi-solid, yield (0.042 g, 54%), ^{1}H NMR (500 MHz, CD_3OD) δ 7.75 (dd, J = 3.7, 1.2 Hz, 1H), 7.65 (dd, J = 5.0, 1.2 Hz, 1H), 7.36 - 7.25 (m, 2H), 7.26 - 7.19 (m, 2H), 7.14 (dd, J = 5.0, 3.8 Hz, 1H), 3.05 - 2.92 (m, 1H), 2.92 - 2.80 (m, 1H), 1.85 (s,

3H), ^{13}C NMR[1], ^{19}F NMR[1], HRMS (ESI), m/z calcd for $C_{15}H_{12}F_3NOS$ $[M+H]^+$: 312.0665, found: 312.0661.

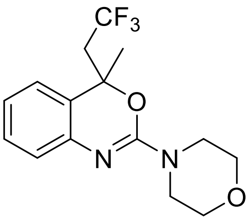

4-methyl-2-morpholino-4-(2,2,2-trifluoroethyl)-4H-benzo[d][1,3]oxazine (2r): White semi-solid, yield (0.047 g, 60%), ^1H NMR (500 MHz, CD_3OD) δ 6.98 - 6.86 (m, 2H), 6.71 (td, J = 7.6, 1.2 Hz, 1H), 6.67 (d, J = 7.9 Hz, 1H), 3.46 - 3.38 (m, 4H), 3.35 - 3.28 (m, 4H), 2.72 - 2.55 (m, 1H), 2.56 - 2.44 (m, 1H), 1.51 (s, 3H), ^{13}C NMR[2], ^{19}F NMR[2], HRMS (ESI), m/z calcd for $C_{15}H_{17}F_3N_2O_2$ $[M+H]^+$: 315.1315, found: 315.1316.

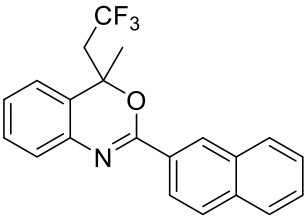

4-methyl-2-(naphthalen-2-yl)-4-(2,2,2-trifluoroethyl)-4H-benzo[d][1,3]oxazine (2s): White semi-solid, yield (0.060 g, 67%), ^1H NMR (500 MHz, CD_3OD) δ 8.61 (s, 1H), 8.21 (dd, J = 8.6, 1.7 Hz, 1H), 8.05 - 7.96 (m, 1H), 7.93 (t, J = 8.8 Hz, 2H), 7.65 - 7.50 (m, 2H), 7.46 - 7.31 (m, 3H), 7.30 - 7.22 (m, 1H), 3.15 - 3.02 (m, 1H), 3.02 - 2.91 (m, 1H), 1.92 (s, 3H), ^{13}C NMR[1], ^{19}F NMR[1], HRMS (ESI), m/z calcd for $C_{21}H_{16}F_3NO$ $[M+H]^+$: 356.1257, found: 356.1258.

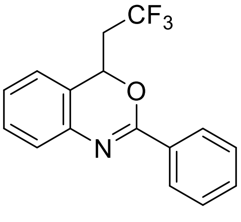

2-phenyl-4-(2,2,2-trifluoroethyl)-4H-benzo[d][1,3]oxazine (2t): Pale yellow semi-solid, yield (0.045 g, 62%), ^1H NMR (500 MHz, CD_3OD) δ 8.13 - 8.06 (m, 2H), 7.58 - 7.47 (m, 1H), 7.45 (dd, J = 8.4, 6.9 Hz, 2H), 7.35 (td, J = 7.6, 1.5 Hz, 1H), 7.29 - 7.22 (m, 2H), 7.17 (dd, J = 7.6, 1.4 Hz, 1H), 5.89 (dd, J = 9.9, 2.9 Hz, 1H), 2.91 - 2.78 (m, 1H), 2.78 - 2.68 (m, 1H), ^{13}C NMR[1], ^{19}F NMR[1], HRMS (ESI), m/z calcd for $C_{16}H_{12}F_3NO$ $[M+H]^+$: 292.0944, found: 292.0948.

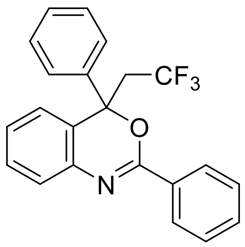

2,4-diphenyl-4-(2,2,2-trifluoroethyl)-4H-benzo[d][1,3]oxazine (2u): White solid, yield (0.049 g, 53%), ^{1}H NMR (500 MHz, CD_3OD) δ 8.27 - 8.19 (m, 2H), 7.62 - 7.53 (m, 1H), 7.52 - 7.46 (m, 3H), 7.42 - 7.35 (m, 3H), 7.34 - 7.20 (m, 5H), 3.52 (q, J = 10.2 Hz, 2H), ^{13}C NMR[1], ^{19}F NMR[1], HRMS (ESI), m/z calcd for $C_{22}H_{16}F_3NO$ $[M+H]^+$: 368.1257, found: 368.1255.

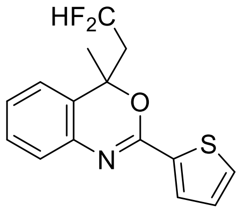

4-(2,2-difluoroethyl)-4-methyl-2-(thiophen-2-yl)-4H-benzo[d][1,3]oxazine (2v): White solid, yield (0.023 g, 31%), ^{1}H NMR (500 MHz, CD_3OD_SPE) δ 7.79 (dd, J = 3.8, 1.2 Hz, 1H), 7.67 (dd, J = 4.9, 1.2 Hz, 1H), 7.40 - 7.30 (m, 1H), 7.30 - 7.19 (m, 3H), 7.16 (dd, J = 5.0, 3.8 Hz, 1H), 6.05 (tdd, J = 55.8, 5.2, 4.0 Hz, 1H), 2.72 - 2.59 (m, 1H), 2.59 - 2.47 (m, 1H), 1.78 (s, 3H), ^{13}C NMR[4], ^{19}F NMR[4], HRMS (ESI): calcd for $C_{15}H_{13}F_2NOS$ $[M+H]^+$: 294.0759, found: 294.0763.

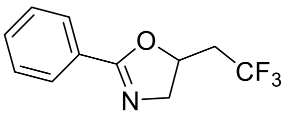

2-phenyl-5-(2,2,2-trifluoroethyl)-4,5-dihydrooxazole (4a): White solid, yield (0.034 g, 59%), ^{1}H NMR (400 MHz, CD_3OD) δ 7.94 - 7.86 (m, 2H), 7.59 - 7.51 (m, 1H), 7.49 - 7.41 (m, 2H), 5.13 - 5.01 (m, 1H), 4.24 (dd, J = 14.7, 9.7 Hz, 1H), 3.78 (dd, J = 14.7, 7.4 Hz, 1H), 2.82 - 2.55 (m, 2H), ^{13}C NMR[3], ^{19}F NMR[3], HRMS (ESI), m/z calcd for $C_{11}H_{10}F_3NO$ $[M+H]^+$: 230.0787, found: 230.0785.

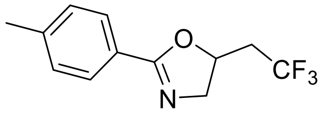

2-(p-tolyl)-5-(2,2,2-trifluoroethyl)-4,5-dihydrooxazole (4b): White solid, yield (0.020 g, 33%), ^{1}H NMR (400 MHz, CD_3OD) δ 7.83 - 7.69 (m, 2H), 7.28 (d, J = 7.9 Hz, 2H), 5.11 - 4.99 (m, 1H), 4.22 (dd, J = 14.6, 9.6 Hz, 1H), 3.76 (dd, J = 14.6, 7.4

Hz, 1H), 2.77 - 2.56 (m, 2H), 2.39 (s, 3H), ^{13}C NMR[3], ^{19}F NMR[3], HRMS (ESI), m/z calcd for $C_{12}H_{12}F_3NO$ $[M+H]^+$: 244.0944, found: 244.0948.

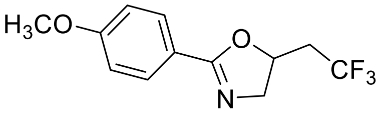

2-(4-methoxyphenyl)-5-(2,2,2-trifluoroethyl)-4,5-dihydrooxazole (4c): White solid, yield (0.030 g, 46%), ^{1}H NMR (500 MHz, CD_3OD) δ 7.95 - 7.72 (m, 2H), 7.10 - 6.76 (m, 2H), 5.09 - 4.99 (m, 1H), 4.20 (dd, J = 14.5, 9.6 Hz, 1H), 3.85 (s, 3H), 3.74 (dd, J = 14.5, 7.3 Hz, 1H), 2.75 - 2.54 (m, 2H), ^{13}C NMR[3], ^{19}F NMR[3], HRMS (ESI), m/z calcd for $C_{12}H_{12}F_3NO_2$ $[M+H]^+$: 260.0893, found: 260.0892.

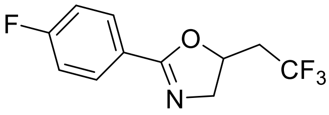

2-(4-fluorophenyl)-5-(2,2,2-trifluoroethyl)-4,5-dihydrooxazole (4d): White solid, yield (0.040 g, 65%), ^{1}H NMR (500 MHz, CD_3OD) δ 7.93 - 7.85 (m, 2H), 7.22 - 7.14 (m, 2H), 5.99 - 5.87 (m, 1H), 5.23 (dq, J = 17.2, 1.7 Hz, 1H), 5.13 (dq, J = 10.2, 1.5 Hz, 1H), 3.99 (dt, J = 5.6, 1.7 Hz, 2H), ^{13}C NMR[3], ^{19}F NMR[3], HRMS (ESI), m/z calcd for $C_{11}H_9F_4NO$ $[M+H]^+$: 248.0693, found: 248.0692.

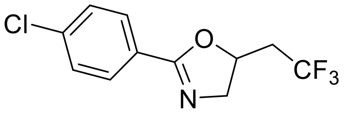

2-(4-chlorophenyl)-5-(2,2,2-trifluoroethyl)-4,5-dihydrooxazole (4e): White solid, yield (0.049 g, 74%), ^{1}H NMR (500 MHz, CD_3OD) δ 8.08 - 7.74 (m, 2H), 7.60 - 7.32 (m, 2H), 5.13 - 4.98 (m, 1H), 4.24 (dd, J = 14.8, 9.7 Hz, 1H), 3.78 (dd, J = 14.8, 7.4 Hz, 1H), 2.88 - 2.51 (m, 2H), ^{13}C NMR[3], ^{19}F NMR[3], HRMS (ESI), m/z calcd for $C_{11}H_9ClF_3NO$ $[M+H]^+$: 264.0398, found: 264.0401.

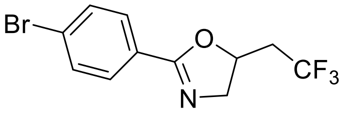

2-(4-bromophenyl)-5-(2,2,2-trifluoroethyl)-4,5-dihydrooxazole (4f): White solid, yield (0.053 g, 69%), ^{1}H NMR (500 MHz, $CDCl_3$) δ 7.80 (d, J = 8.3 Hz, 2H), 7.56 (d, J = 8.2 Hz, 2H), 5.03 - 4.93 (m, 1H), 4.26 (dd, J = 15.0, 9.6 Hz, 1H), 3.78 (dd, J = 15.0, 7.3 Hz, 1H), 2.72 - 2.57 (m, 1H), 2.48 - 2.35 (m, 1H), ^{13}C NMR[3], ^{19}F NMR[3], HRMS (ESI), m/z calcd for $C_{11}H_9BrF_3NO$ $[M+H]^+$: 307.9893, found: 307.9895.

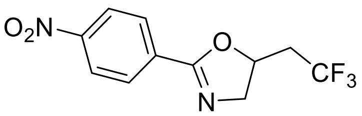

2-(4-nitrophenyl)-5-(2,2,2-trifluoroethyl)-4,5-dihydrooxazole (4g): White solid, yield (0.056 g, 82%), ^{1}H NMR (400 MHz, DMSO) δ 8.47 - 8.23 (m, 2H), 8.21 - 7.91 (m, 2H), 5.15 - 5.02 (m, 1H), 4.24 (dd, J = 15.5, 9.8 Hz, 1H), 3.79 (dd, J = 15.5, 7.4 Hz, 1H), 2.91 - 2.75 (m, 2H), ^{13}C NMR (100 MHz, DMSO) δ 160.5, 149.1, 132.9, 129.0, 126.1 (q, J = 277 Hz, CF_3), 123.9, 73.8 (q, J = 3.0 Hz), 59.8, 37.8 (q, J = 26.9 Hz), ^{19}F NMR (470 MHz, CD_3OD) δ -65.5 (t, J = 10.8 Hz), HRMS (ESI), m/z calcd for $C_{11}H_9F_3N_2O_3$ $[M+H]^+$: 275.0638, found: 275.0636.

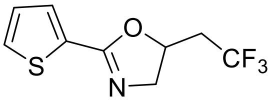

2-(thiophen-2-yl)-5-(2,2,2-trifluoroethyl)-4,5-dihydrooxazole (4h): White solid, yield (0.043 g, 73%), ^{1}H NMR (500 MHz, CD_3OD) δ 7.64 (d, J = 3.7 Hz, 2H), 7.14 (t, J = 4.3 Hz, 1H), 5.11 - 5.01 (m, 1H), 4.25 - 4.16 (m, 1H), 3.79 - 3.71 (m, 1H), 2.78 -- 2.57 (m, 2H), ^{13}C NMR (125 MHz, CD_3OD) δ 161.6, 132.3, 132.1, 130.3, 128.9, 127.2 (q, J = 274 Hz, CF_3), 75.7 (q, J = 3.2 Hz), 60.4, 39.6 (q, J = 28.1 Hz), ^{19}F NMR (470 MHz, CD_3OD) δ -65.5 (t, J = 10.8 Hz), HRMS (ESI), m/z calcd for $C_9H_8F_3NOS$ $[M+H]^+$: 236.0352, found: 236.0352.

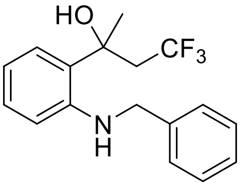

2-(2-(benzylamino)phenyl)-4,4,4-trifluorobutan-2-ol (5): White solid, yield (0.316 g, 78%), ^{1}H NMR (500 MHz, CD_3OD) δ 7.38 (d, J = 7.1 Hz, 2H), 7.31 (t, J = 7.6 Hz, 2H), 7.22 (t, J = 7.3 Hz, 1H), 7.14 (dd, J = 7.8, 1.5 Hz, 1H), 7.05 (ddd, J = 8.6, 7.5, 1.5 Hz, 1H), 6.65 - 6.57 (m, 2H), 4.35 (q, J = 14.6 Hz, 2H), 3.01 - 2.79 (m, 2H), 1.81 (s, 3H), ^{13}C NMR (125 MHz, CD_3OD) δ 146.9, 140.0, 128.8, 128.2, 128.1, 126.9, 126.5, 125.7, 116.1, 111.9, 72.4, 60.4, 41.6 (d, J = 25.7 Hz), 26.7, ^{19}F NMR (470 MHz, CD_3OD) δ 61.5 (t, J = 11.4Hz), HRMS (ESI), m/z calcd for $C_{17}H_{18}F_3NO$ $[M+H]^+$: 310.1418, found: 310.1415.

4. ^{1}H NMR, ^{13}C NMR and ^{19}F NMR Spectra

4-methyl-2-phenyl-4-(2, 2, 2-trifluoromethyl)[d][1,3]oxazine (2a):

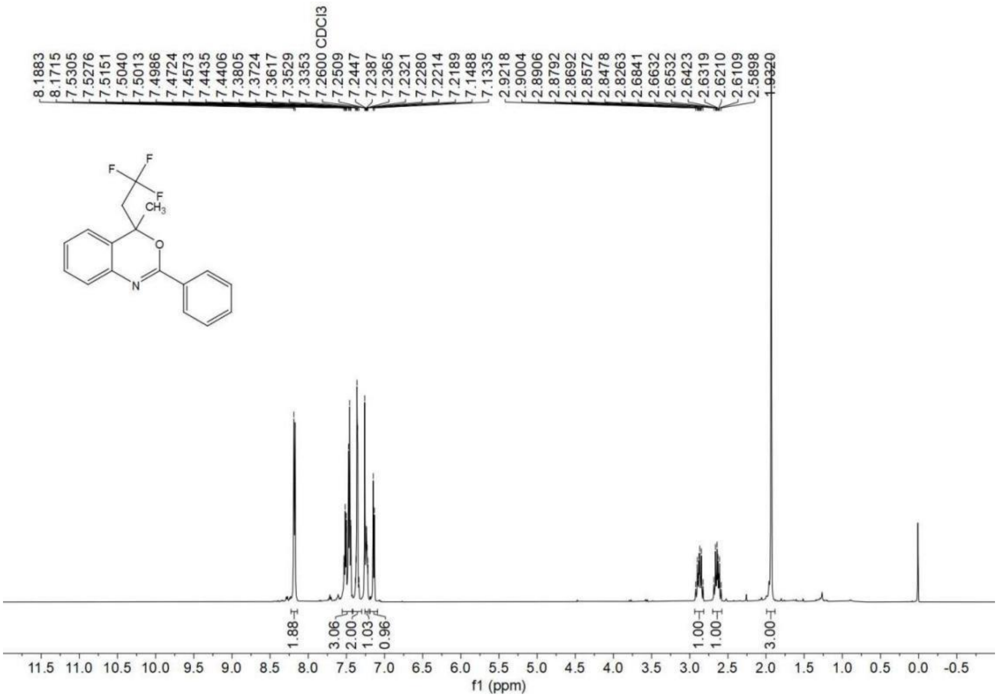

^{1}H NMR Spectrum of **2a** (500 MHz, $CDCl_3$)

4-methyl-2-(p-tolyl)-4-(2,2,2-trifluoroethyl)-4H-benzo[*d*][1,3]oxazine (2b):

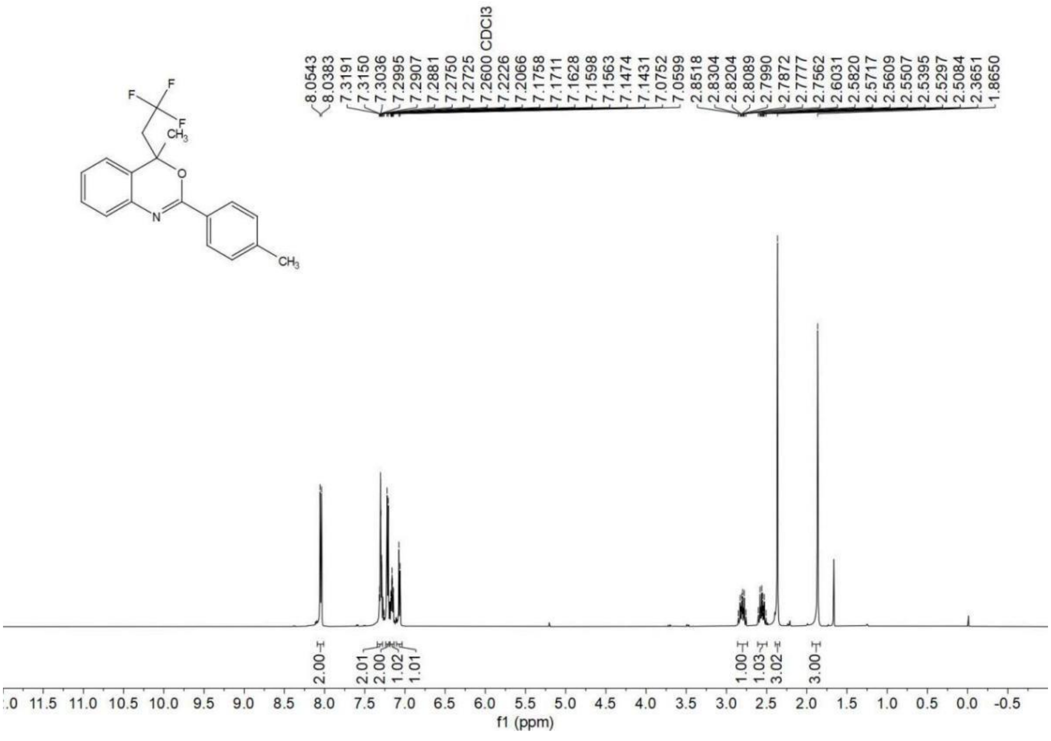

^{1}H NMR Spectrum of **2b** (500 MHz, $CDCl_3$)

2-(4-methoxyphenyl)-4-methyl-4-(2,2,2-trifluoroethyl)-4H-benzo[d][1,3]oxazine (2c):

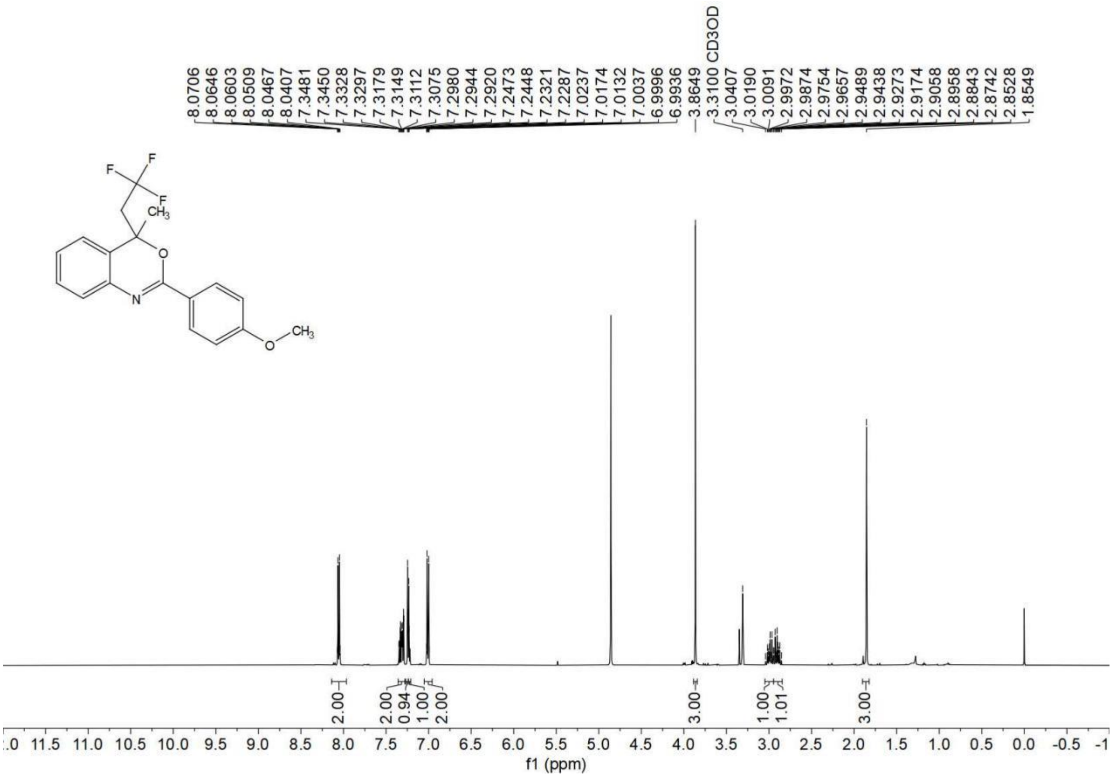

^{1}H NMR Spectrum of **2c** (500 MHz, CD_3OD)

2-(4-fluorophenyl)-4-methyl-4-(2,2,2-trifluoroethyl)-4H-benzo[d][1,3]oxazine (2d):

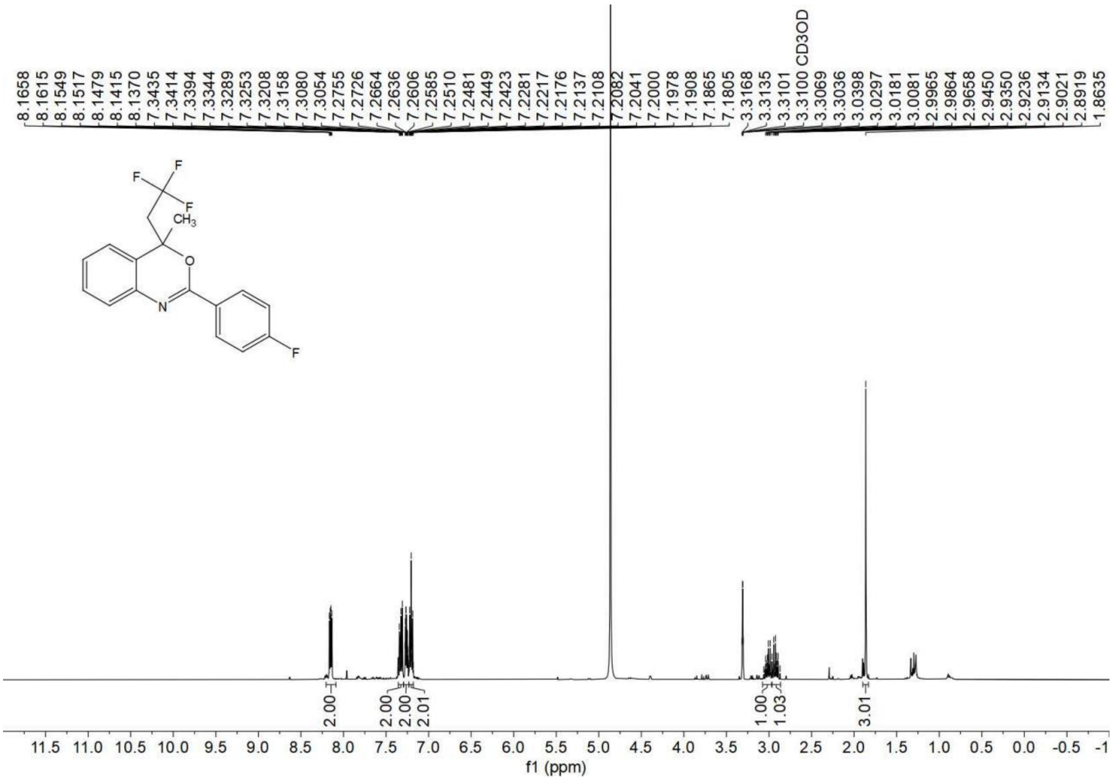

^{1}H NMR Spectrum of **2d** (500 MHz, CD_3OD)

2-(4-chlorophenyl)-4-methyl-4-(2,2,2-trifluoroethyl)-4H-benzo[d][1,3]oxazine(2e):

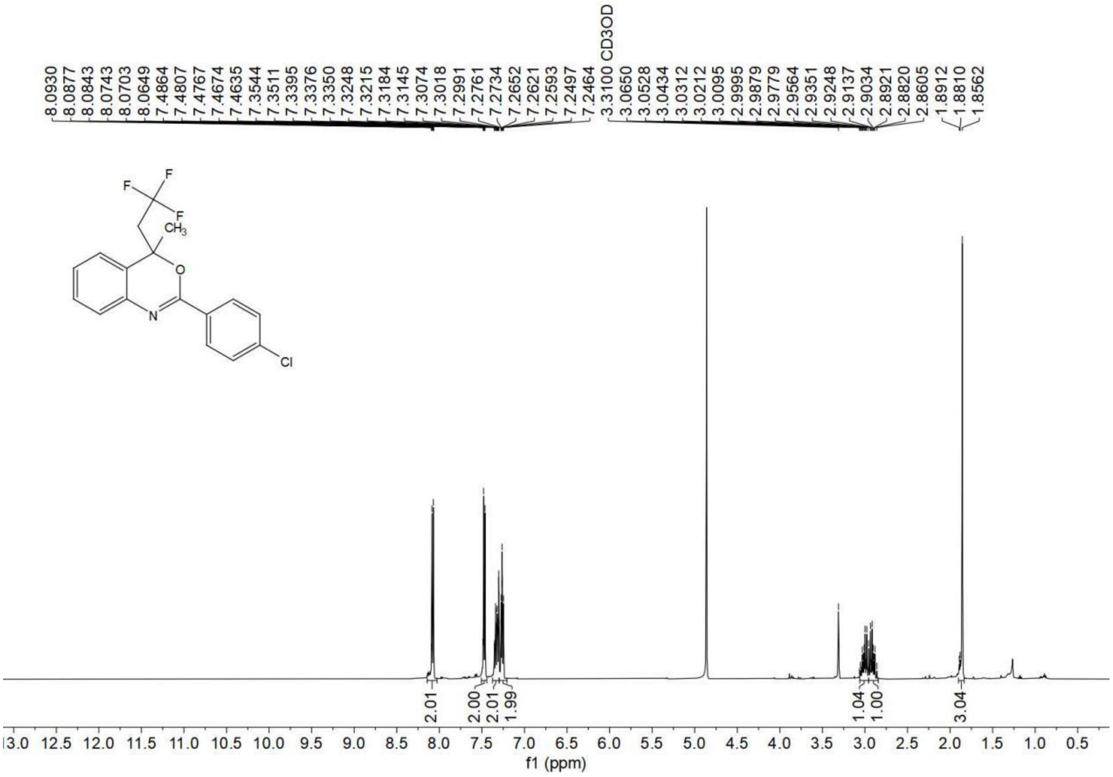

^{1}H NMR Spectrum of **2e** (500 MHz, CD3OD)

2-(4-bromophenyl)-4-methyl-4-(2,2,2-trifluoroethyl)-4H-benzo[d][1,3]oxazine (2f):

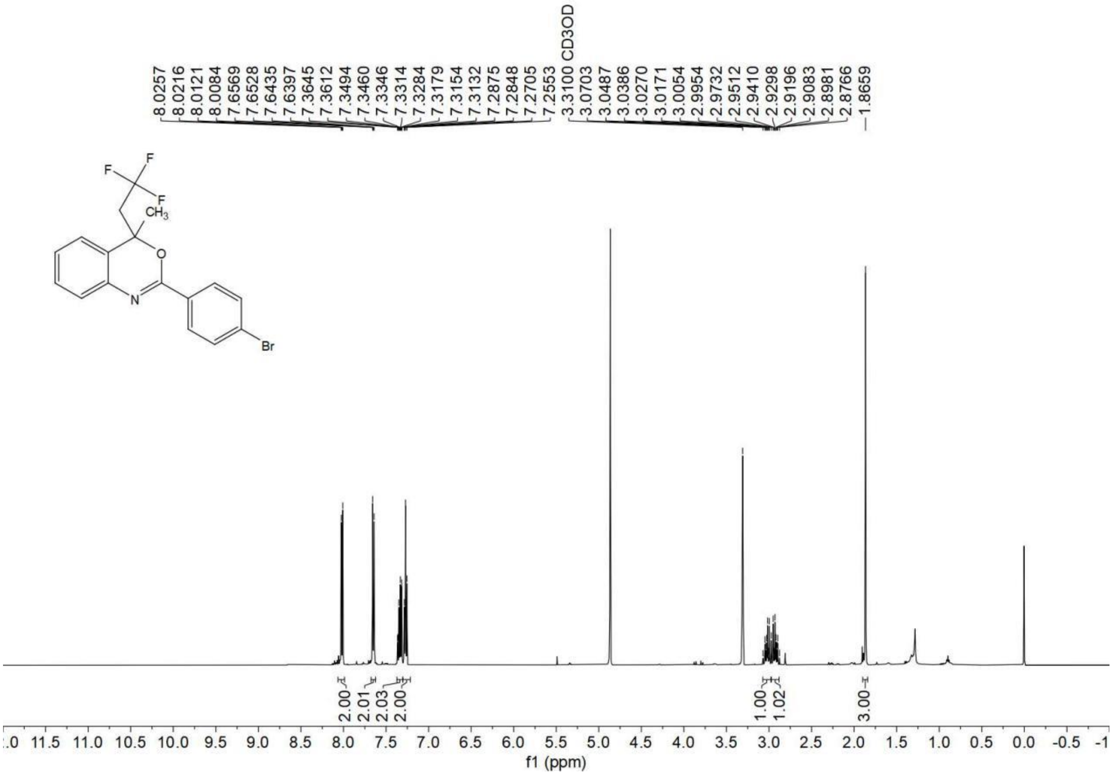

^{1}H NMR Spectrum of **2f** (500 MHz, CD3OD)

4-methyl-4-(2,2,2-trifluoroethl)-2-(4-(trifluoromethyl)phenyl)-4H-benzo[d][1,3]oxazine (2g):

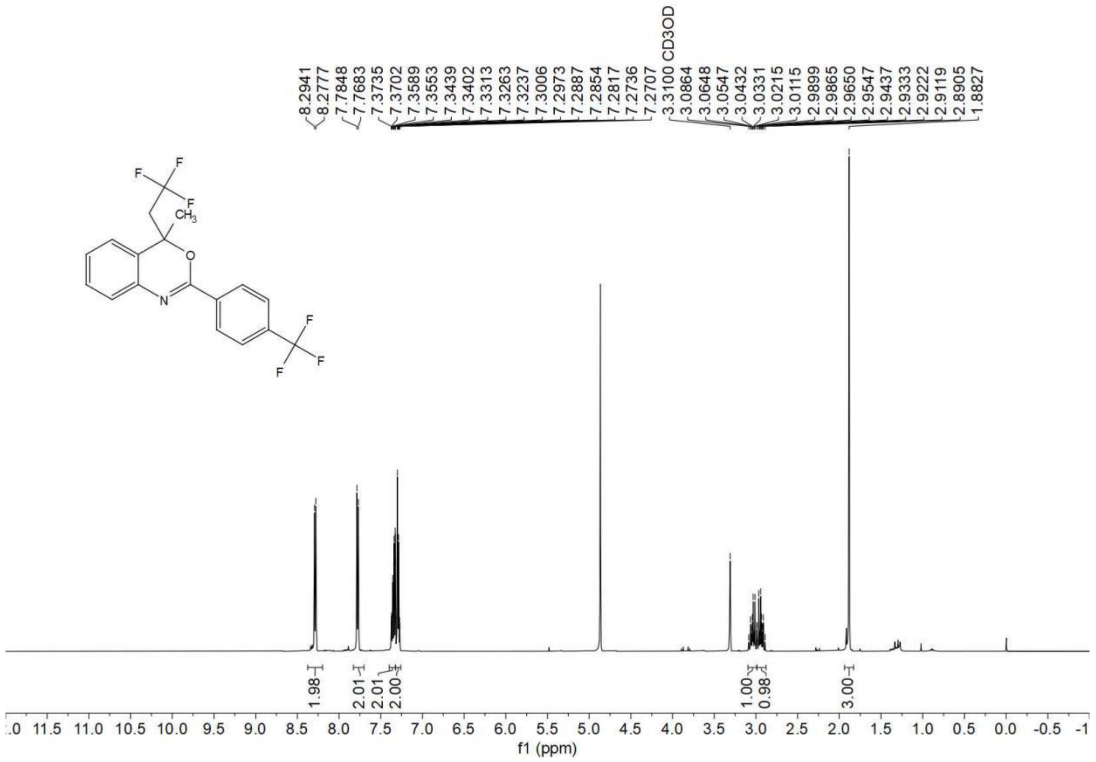

^{1}H NMR Spectrum of **2g** (500 MHz, CD_3OD)

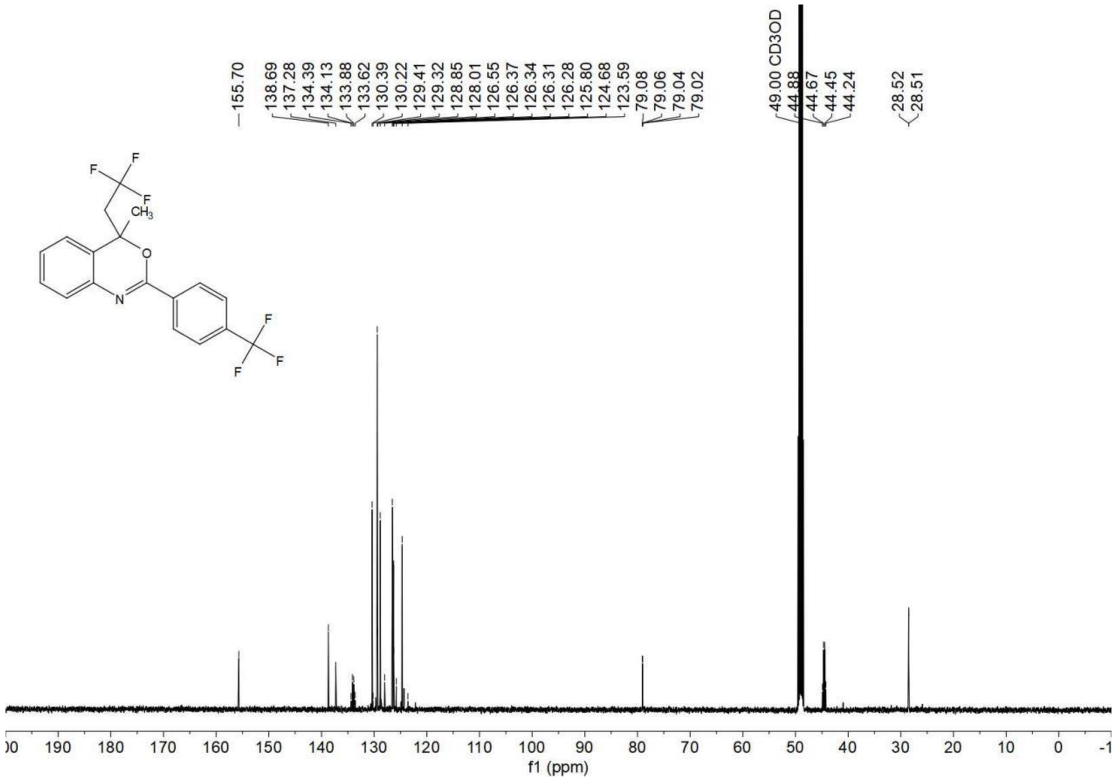

^{13}C NMR Spectrum of **2g** (125 MHz, CD_3OD)

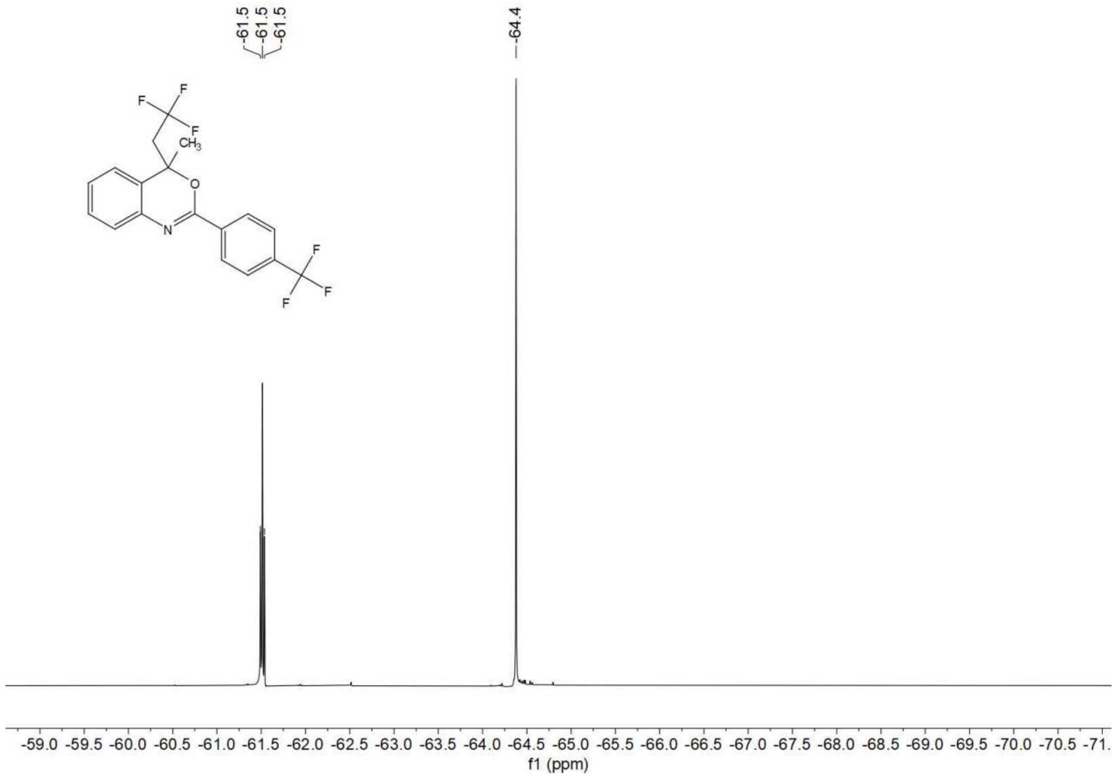

^{19}F NMR Spectrum of **2g** (470 MHz, CD_3OD)

4-methyl-2-(4-nitrophenyl)-4-(2,2,2-trifluoroethyl)-4H-benzo[d][1,3]oxazine (2h):

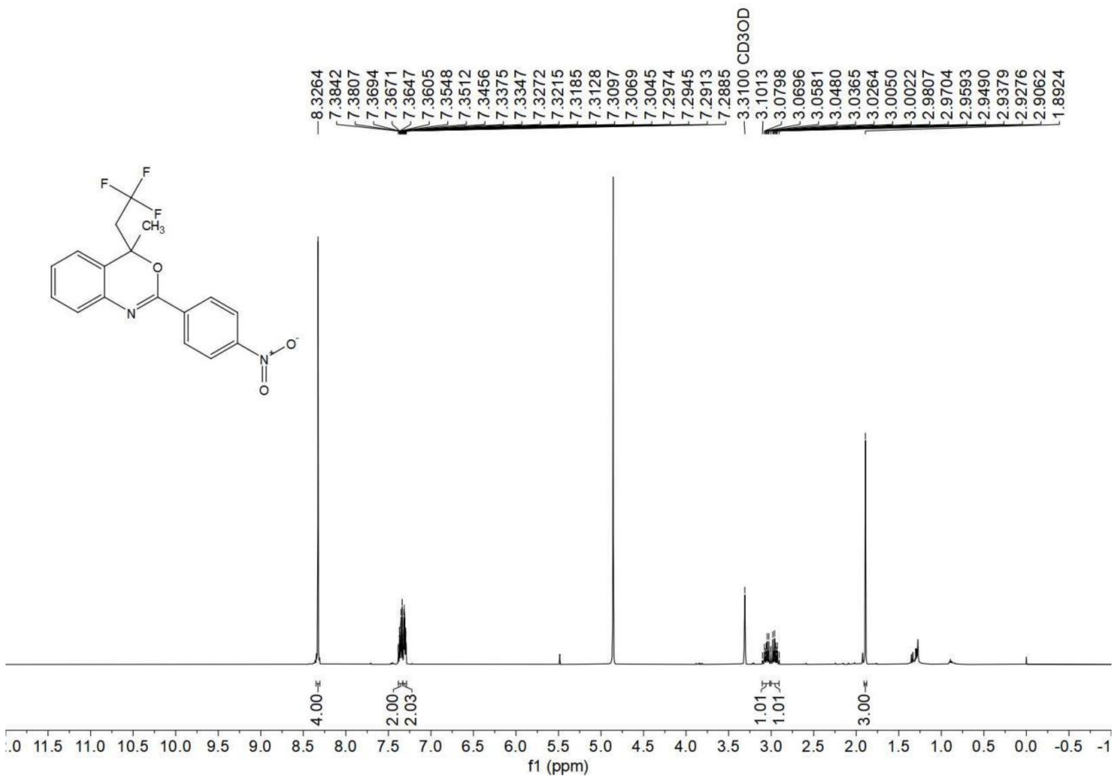

^{1}H NMR Spectrum of **2h** (500 MHz, CD_3OD)

2-(3-chlorophenyl)-4-methyl-4-(2,2,2-trifluoroethyl)-4H-benzo[d][1,3]oxazine (2i):

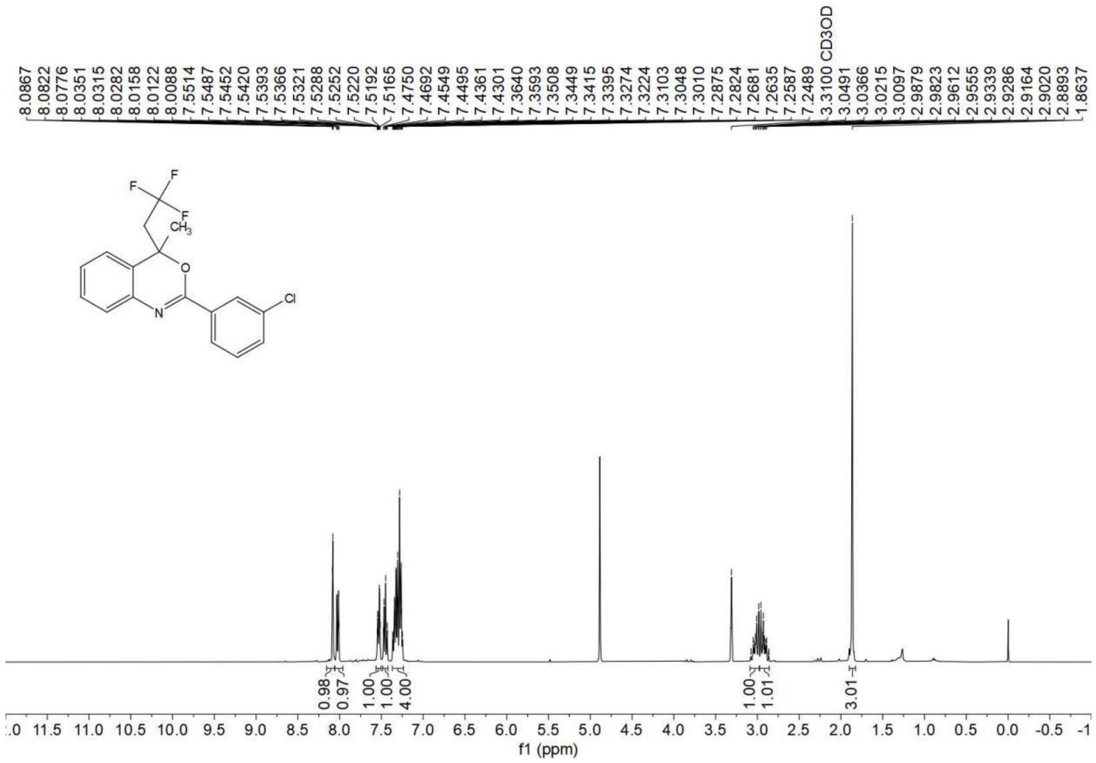

^{1}H NMR Spectrum of **2i** (400 MHz, CD_3OD)

2-(2-chlorophenyl)-4-methyl-4-(2,2,2-trifluoroethyl)-4H-benzo[d][1,3]oxazine (2j):

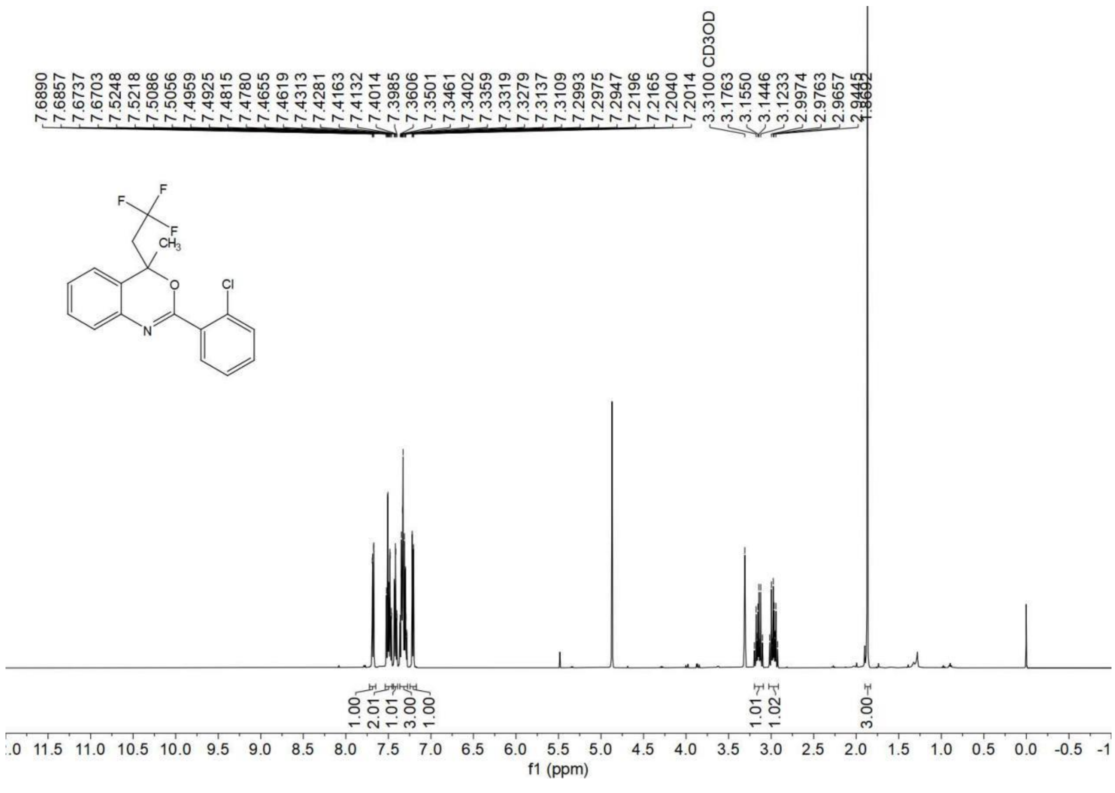

^{1}H NMR Spectrum of **2j** (500 MHz, CD_3OD)

2,4-dimethyl-4-(2,2,2-trifluoroethyl)-4H-benzo[d][1,3]oxazine (2k):

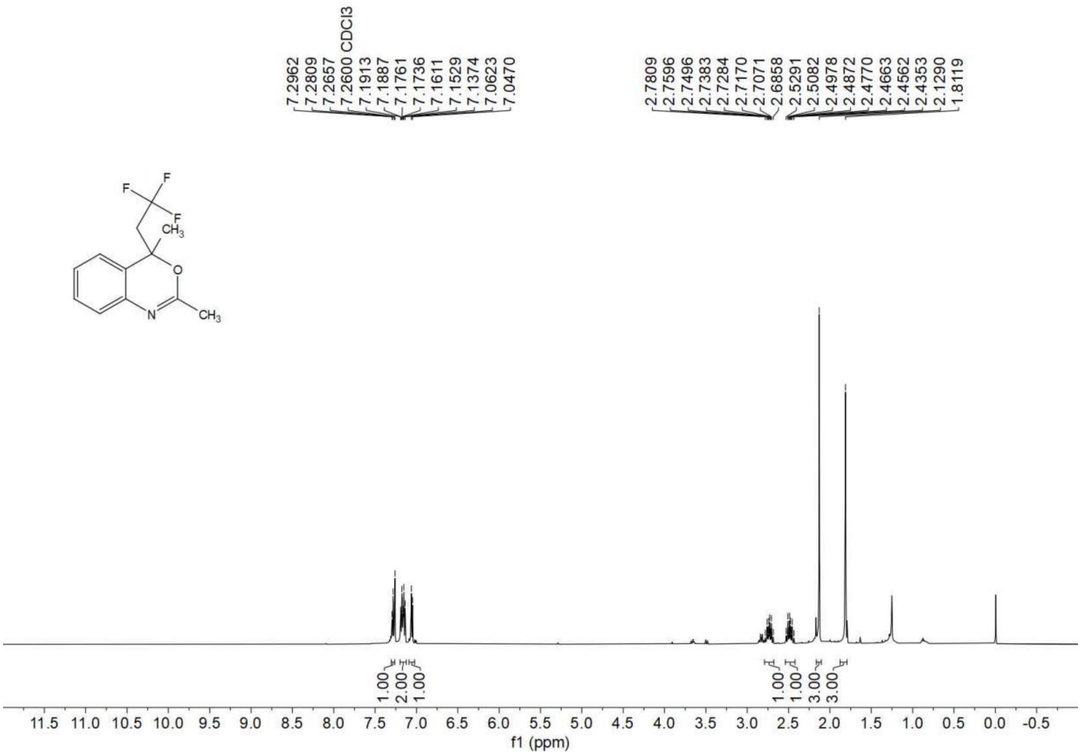

^{1}H NMR Spectrum of **2k** (500 MHz, $CDCl_3$)

2-(tert-butyl)-4-methyl-4-(2,2,2-trifluoroethyl)-4H-benzo[d][1,3]oxazine (2l):

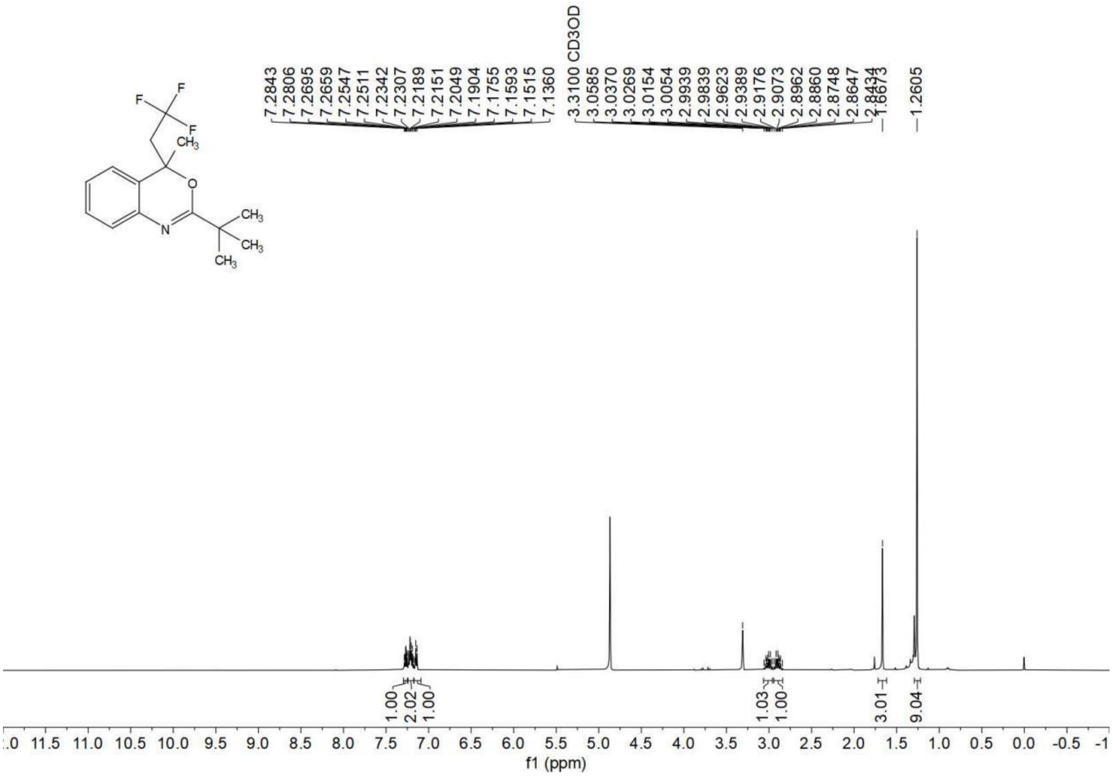

^{1}H NMR Spectrum of **2l** (500 MHz, CD_3OD)

2-cyclopropyl-4-methyl-4-(2,2,2-trifluoroethyl)-4H-benzo[d][1,3]oxazine (2m):

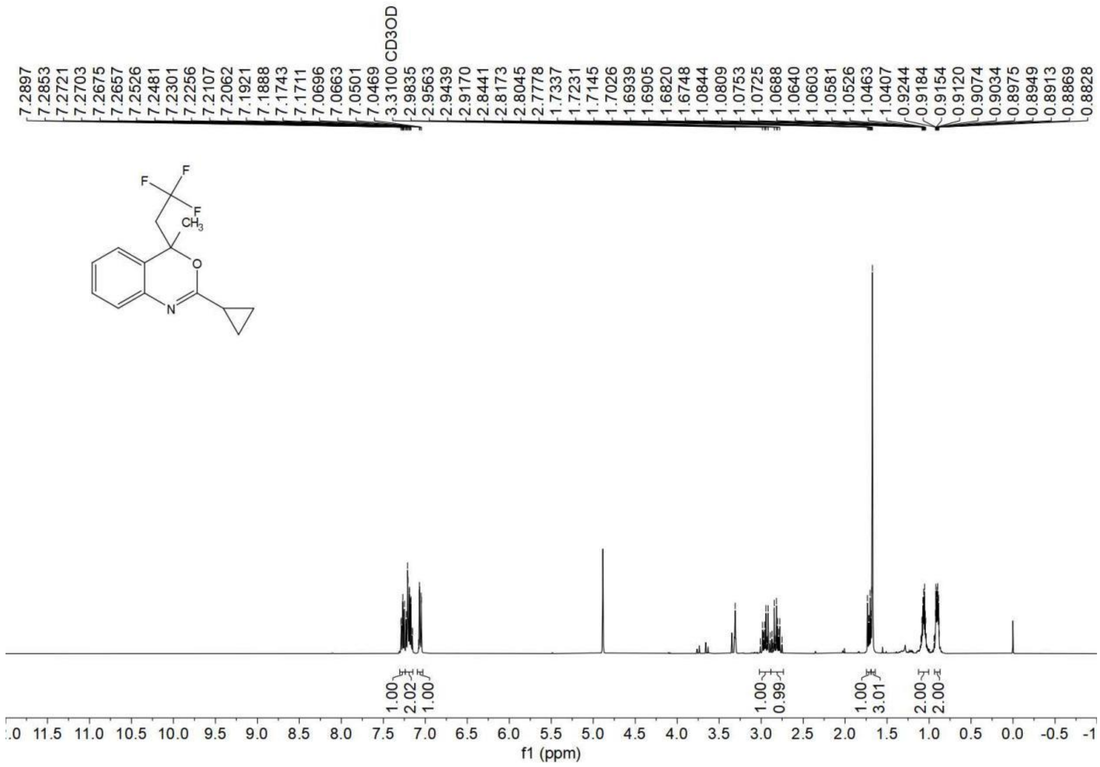

^{1}H NMR Spectrum of **2m** (400 MHz, CD_3OD)

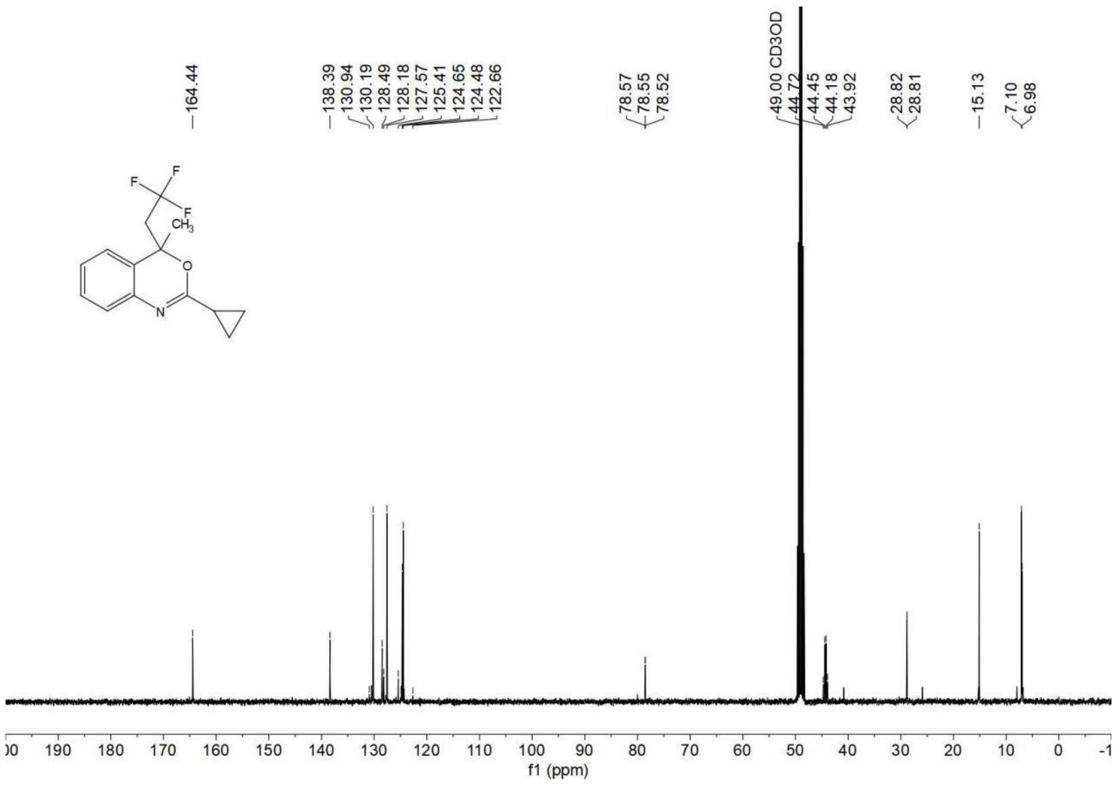

^{13}C NMR Spectrum of **2m** (100 MHz, CD_3OD)

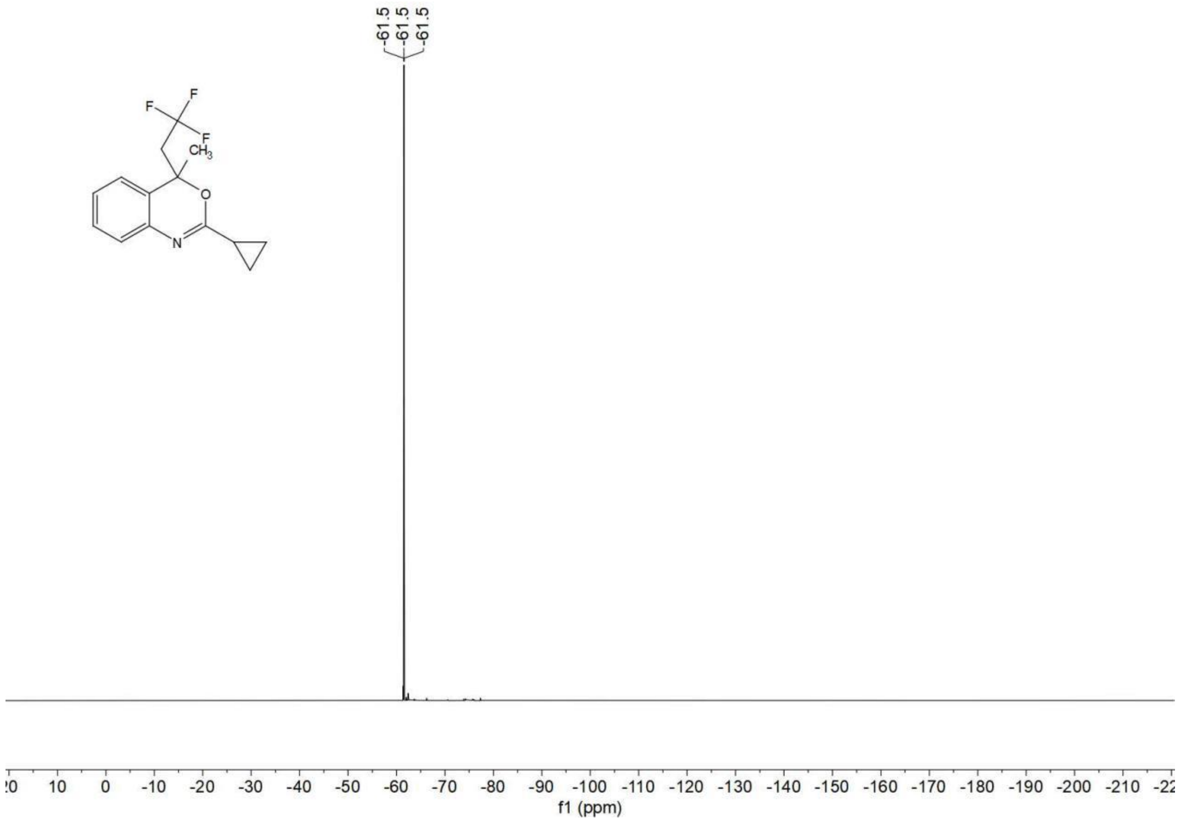

^{19}F NMR Spectrum of **2m** (376 MHz, CD_3OD)

2-cyclohexyl-4-methyl-4-(2,2,2-trifluoroethyl)-4H-benzo[d][1,3]oxazine (2n):

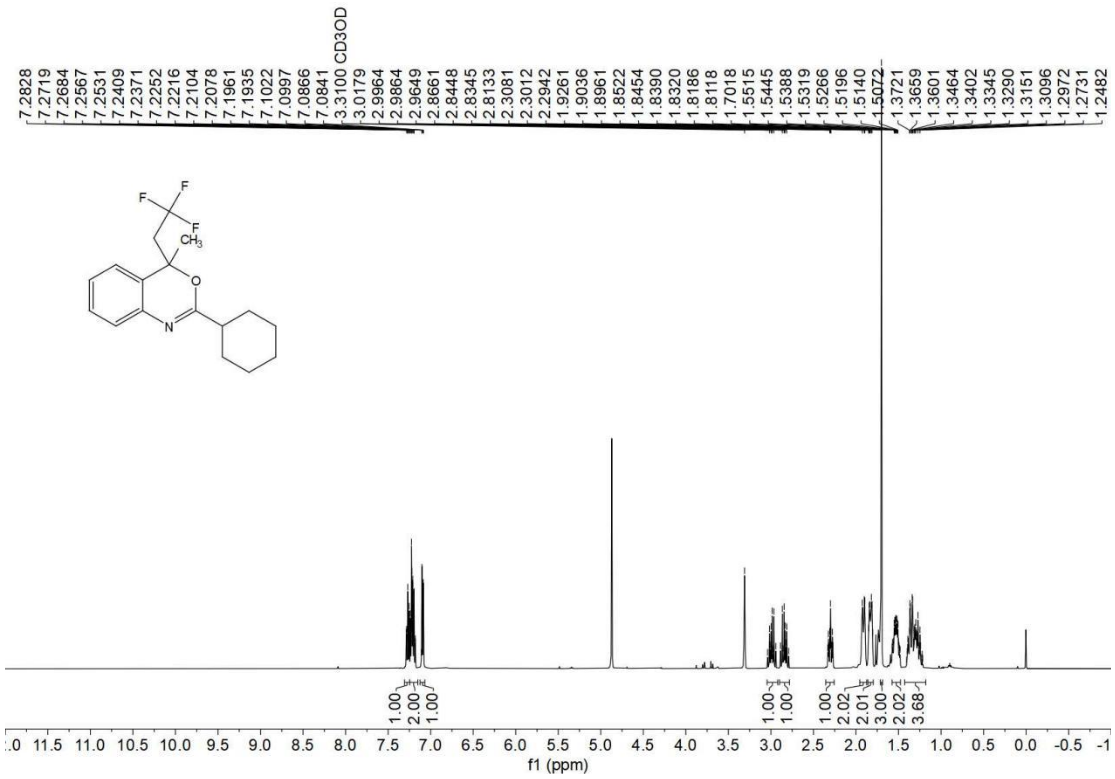

^{1}H NMR Spectrum of **2n** (500 MHz, CD_3OD)

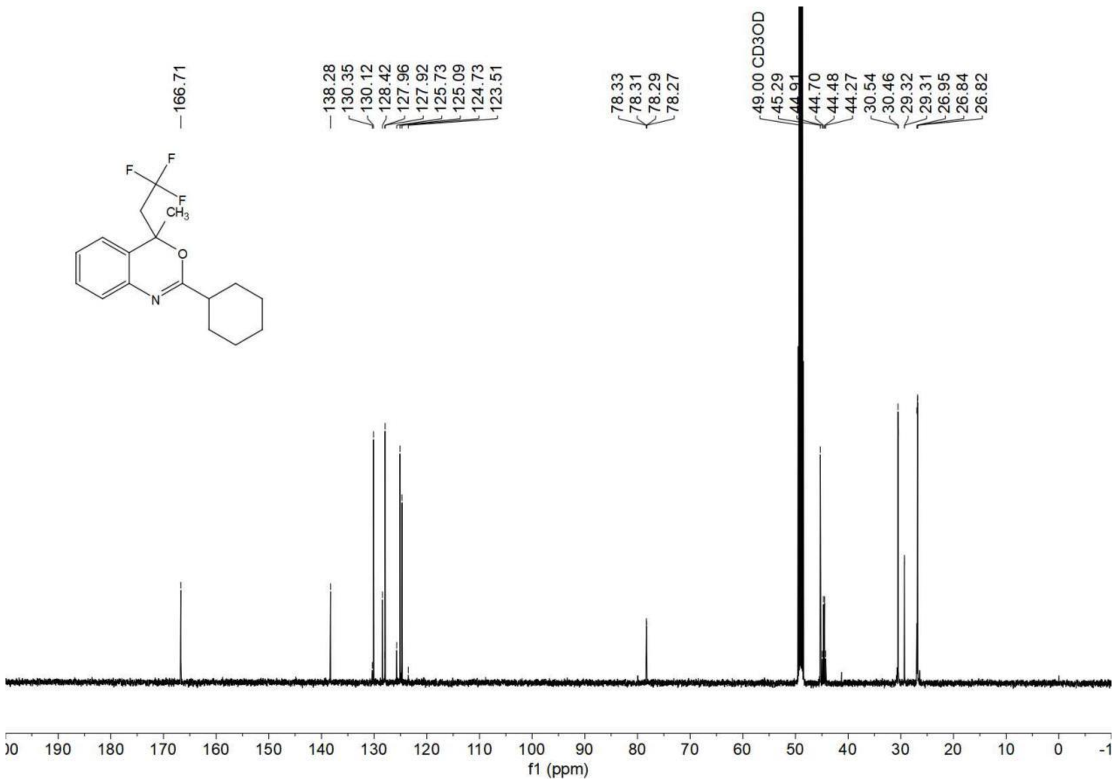

^{13}C NMR Spectrum of **2n** (125 MHz, CD_3OD)

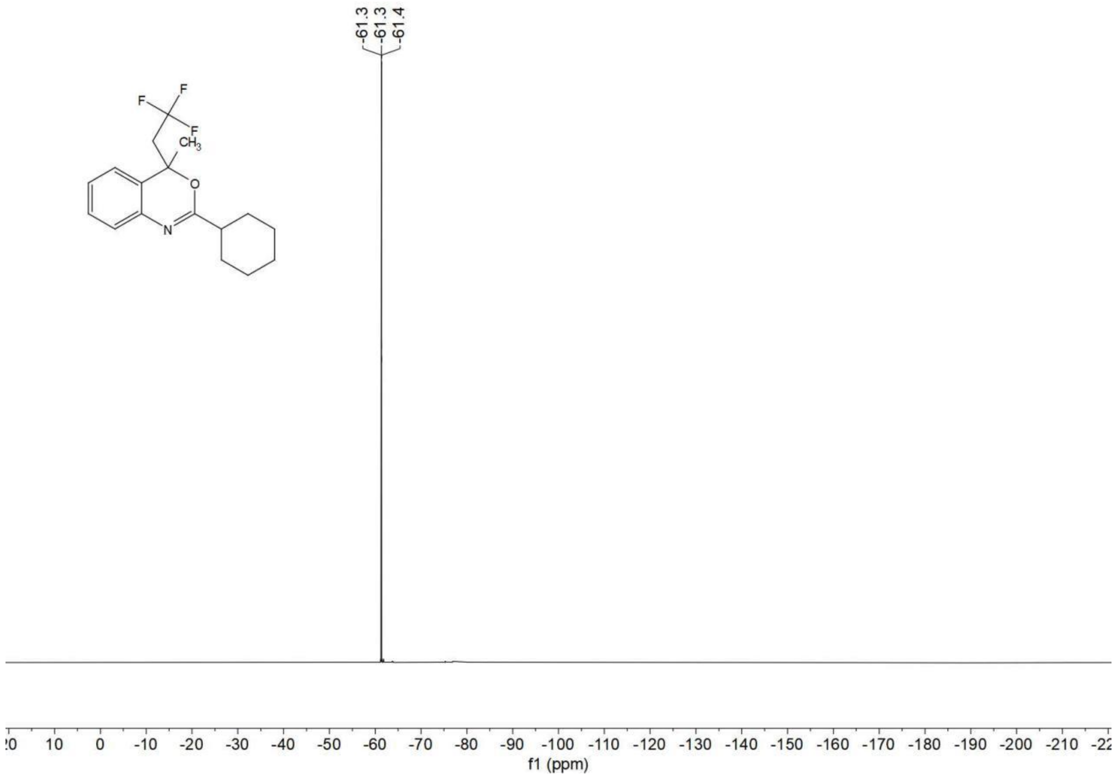

^{19}F NMR Spectrum of **2n** (470 MHz, CD_3OD)

2-(furan-2-yl)-4-methyl-4-(2,2,2-trifluoroethyl)-4H-benzo[d][1,3]oxazine (2o):

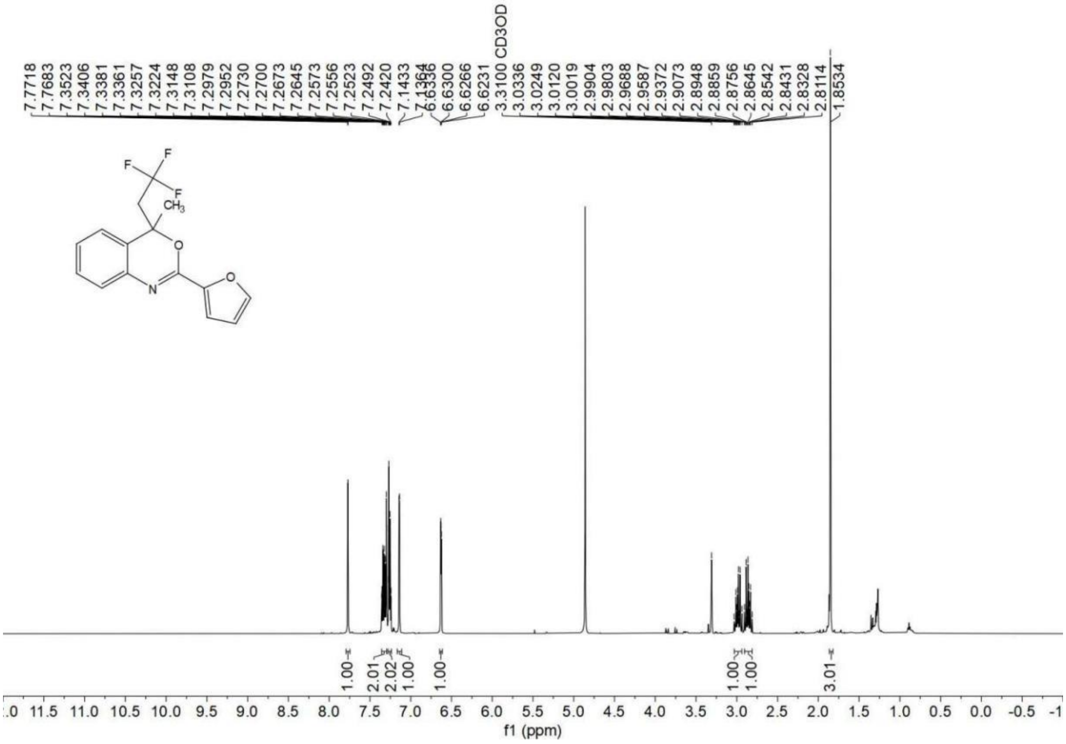

^{1}H NMR Spectrum of **2o** (500 MHz, CD_3OD)

4-methyl-2-(thiophen-2-yl)-4-(2,2,2-trifluoroethyl)-4H-benzo[d][1,3]oxazine (2p):

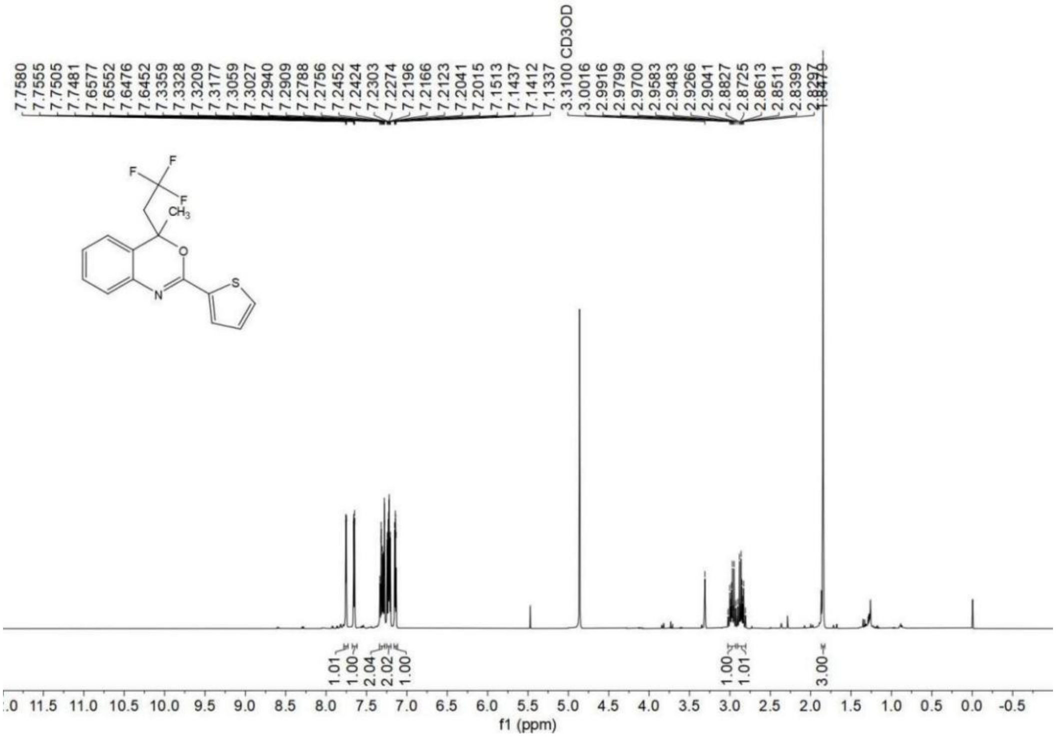

^{1}H NMR Spectrum of **2p** (500 MHz, CD_3OD)

4-methyl-2-morpholino-4-(2,,2-trifluoroethyl)-4H-benzo[d][1,3]oxazine (2r):

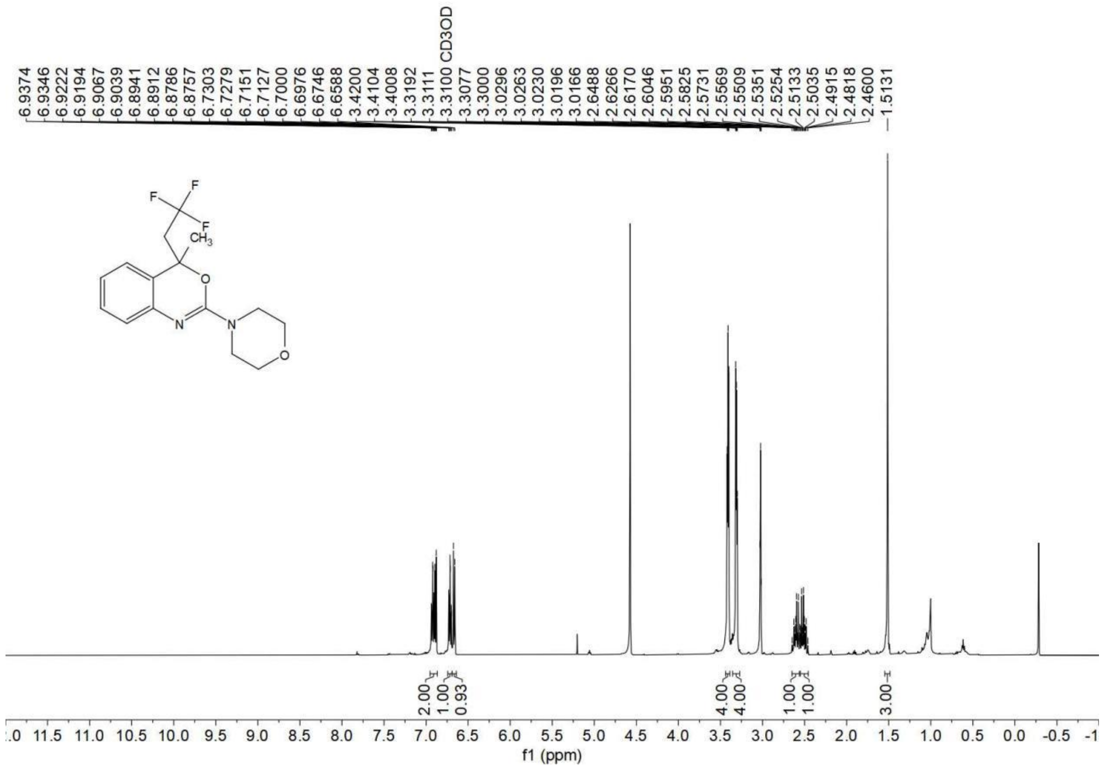

^{1}H NMR Spectrum of **2r** (500 MHz, CD_3OD)

4-methyl-2-(naphthalen-2-yl)-4-(2,2,2-trifluoroethyl)-4H-benzo[d][1,3]oxazine (2s):

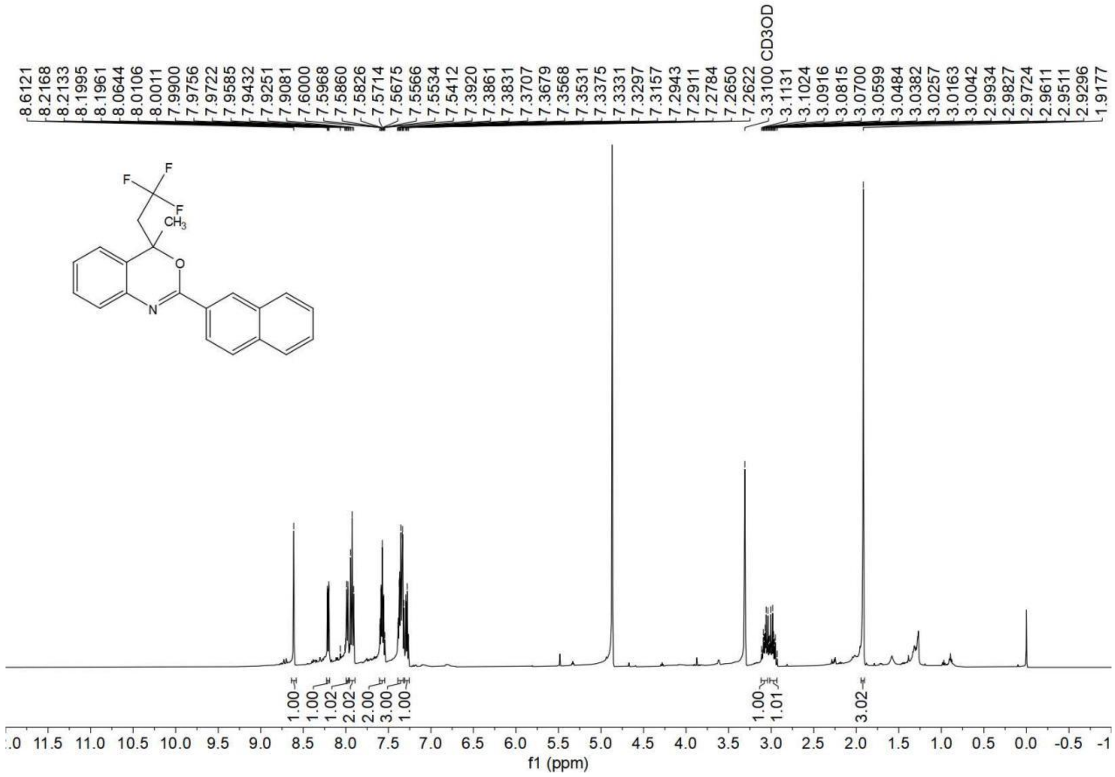

^{1}H NMR Spectrum of **2s** (500 MHz, CD_3OD)

2-phenyl-4-(2,2,2-trifluoroethyl)-4H-benzo[d][1,3]oxazine (2t):

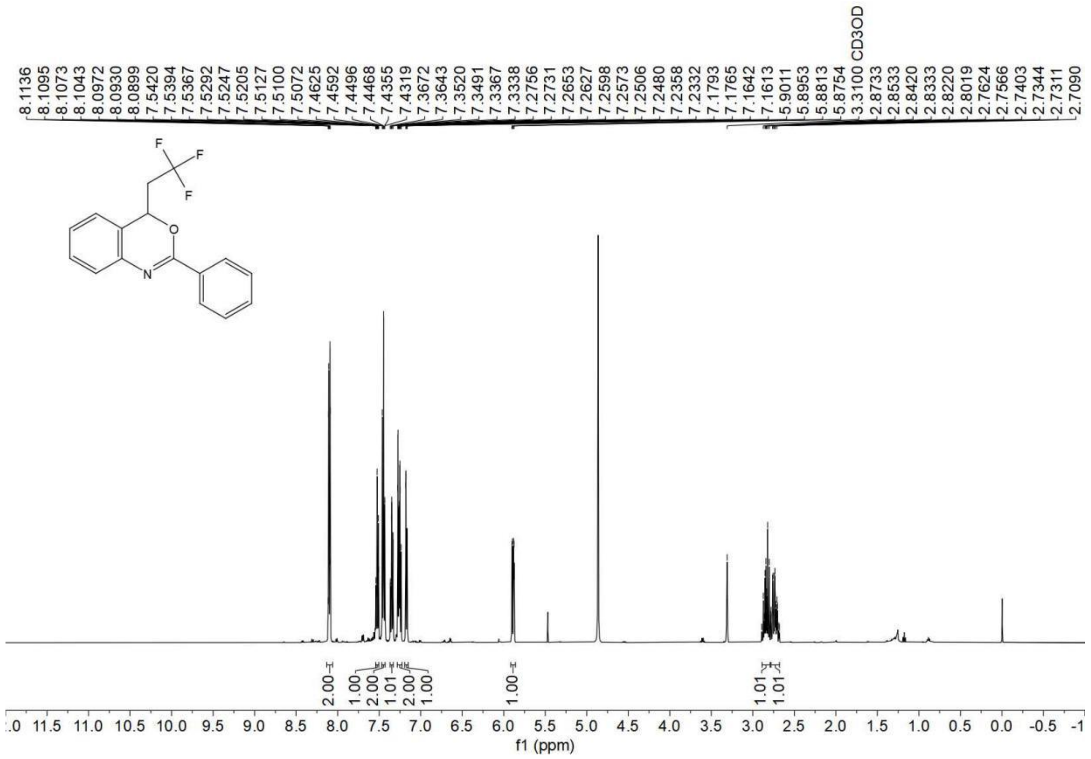

^{1}H NMR Spectrum of **2t** (500 MHz, CD_3OD)

2,4-diphenyl-4-(2,2,2-trifluoroethyl)-4H-benzo[d][1,3]oxazine (2u):

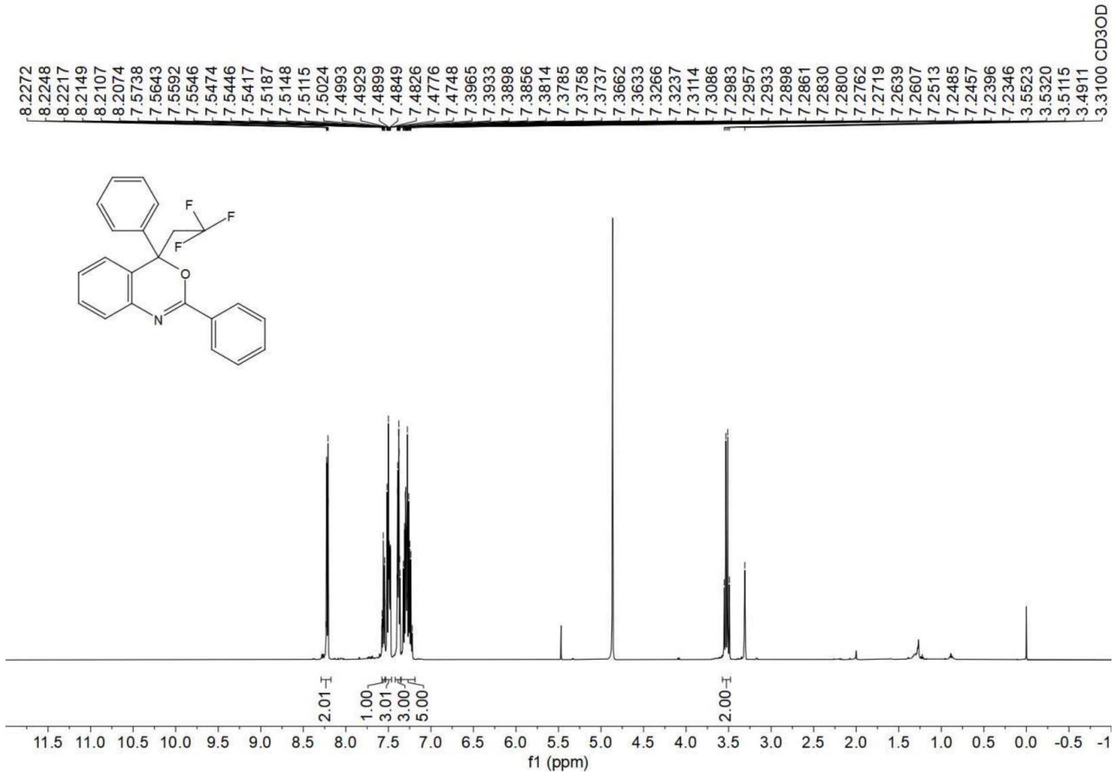

^{1}H NMR Spectrum of **2u** (500 MHz, CD_3OD)

4-(2,2-difluoroethyl)-4-methyl-2-(thiophen-2-yl)-4H-benzo[d][1,3]oxazine (2v):

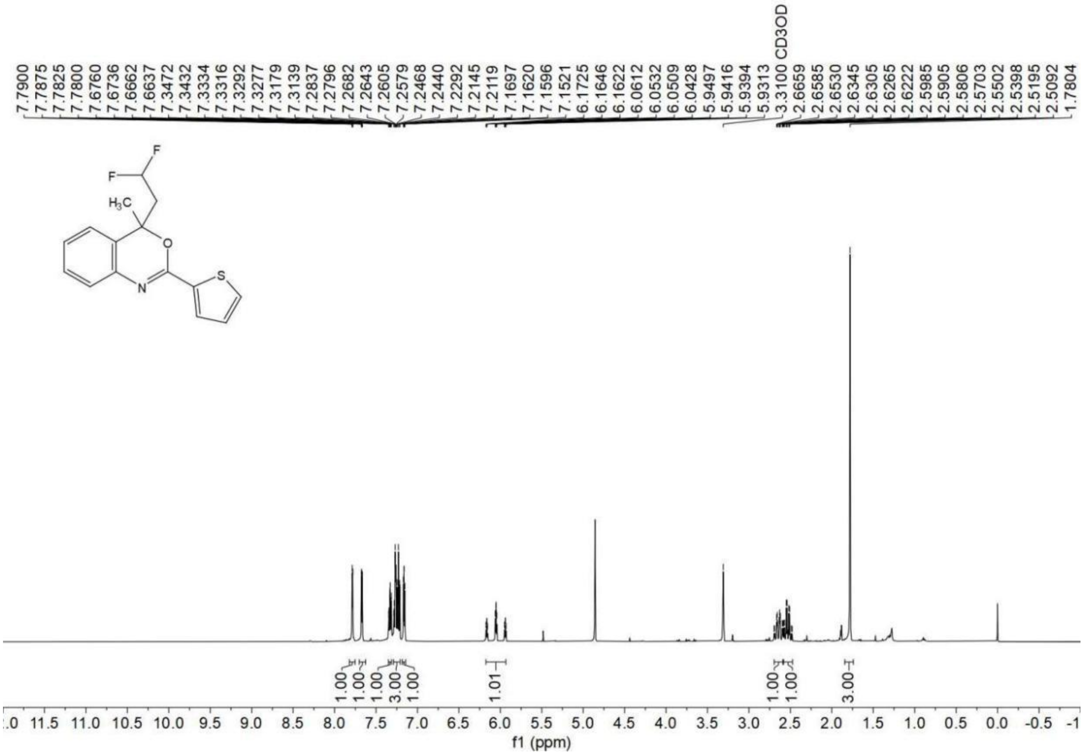

^{1}H NMR Spectrum of **2v** (500 MHz, CD_3OD)

2-phenyl-5-(2,2,2-trifluoroethyl)-4,5-dihydrooxazole (4a):

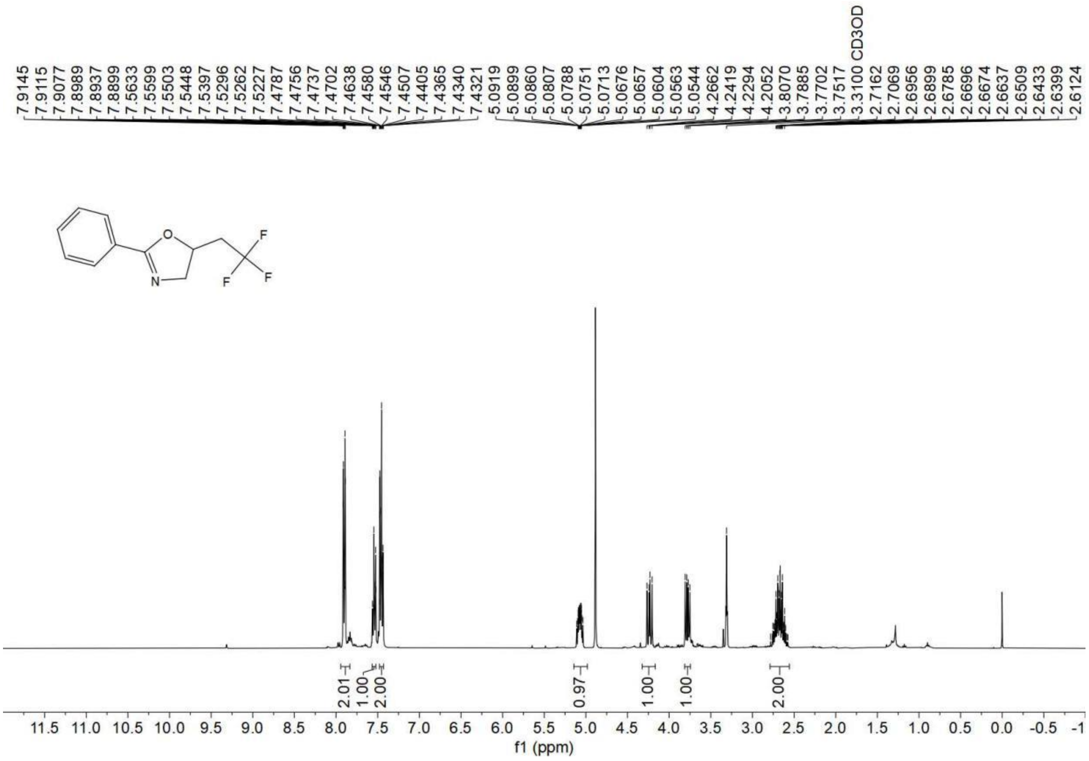

^{1}H NMR Spectrum of **4a** (400 MHz, CD_3OD)

2-(p-tolyl)-5-(2,2,2-trifluoroehyl)-4,5-dihydrooxazole (4b):

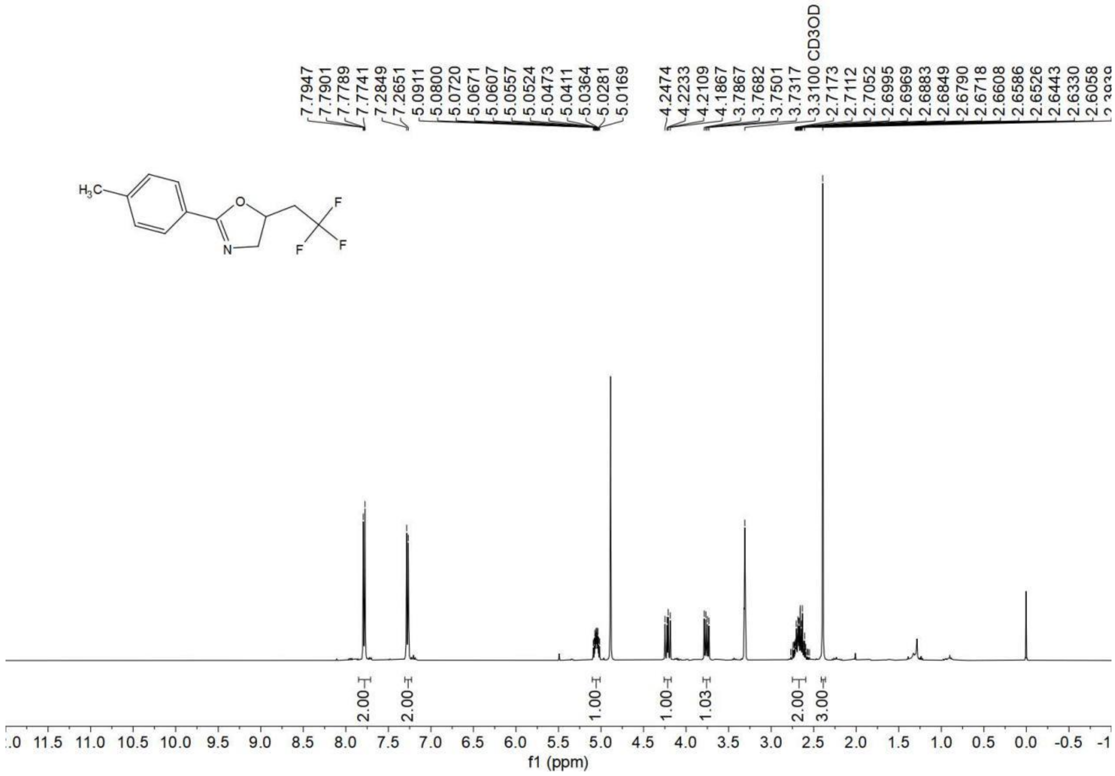

^{1}H NMR Spectrum of **4b** (400 MHz, CD_3OD)

2-(4-methoxyphenyl)-5-(2,22-trifluoroethyl)-4,5-dihydrooxazole (4c):

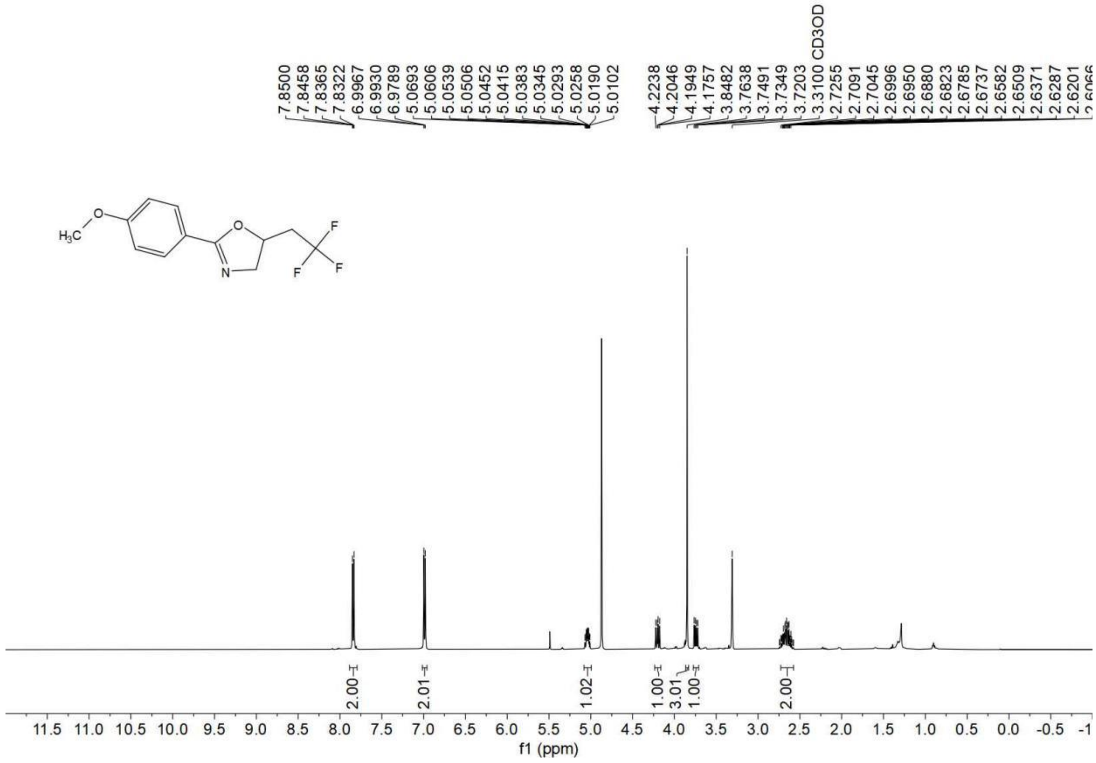

^{1}H NMR Spectrum of **4c** (500 MHz, CD_3OD)

2-(4-fluorophenyl)-5-(2,2,2-trifluoroethyl)-4,5-dihydrooxazole (4d):

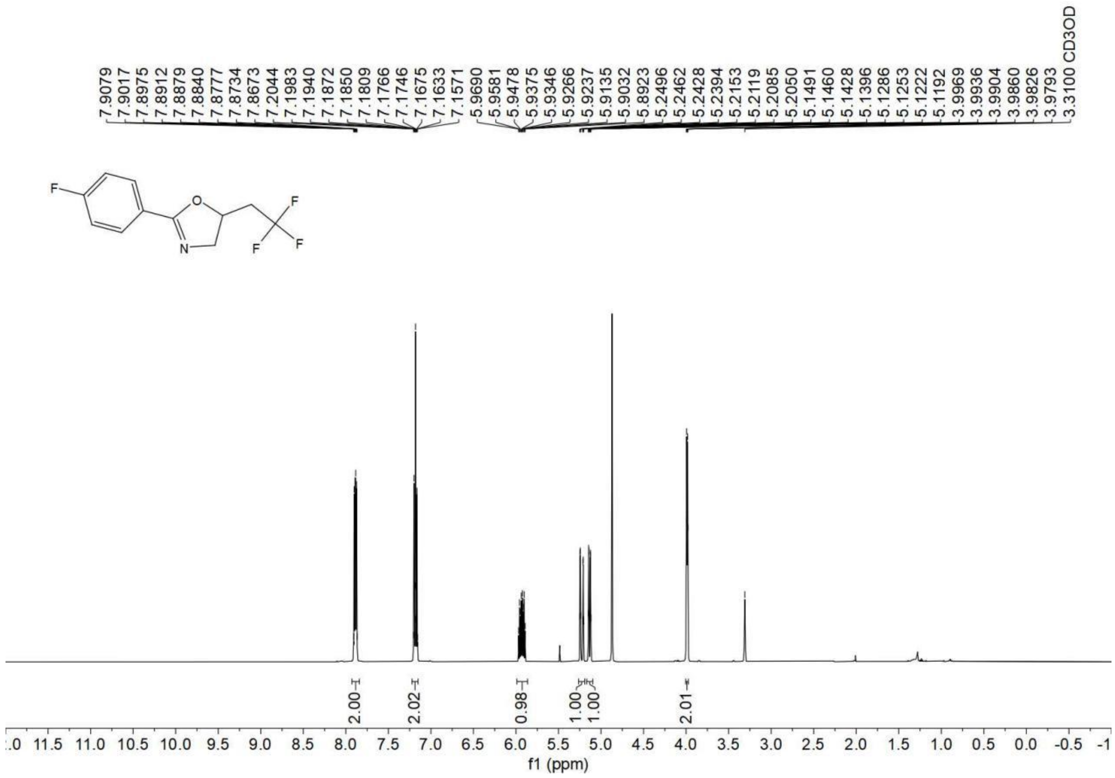

^{1}H NMR Spectrum of **4d** (500 MHz, CD_3OD)

2-(4-chlorophenyl)-5-(2,2,2-trifluoroethyl)-4,5-dihydrooxazole (4e):

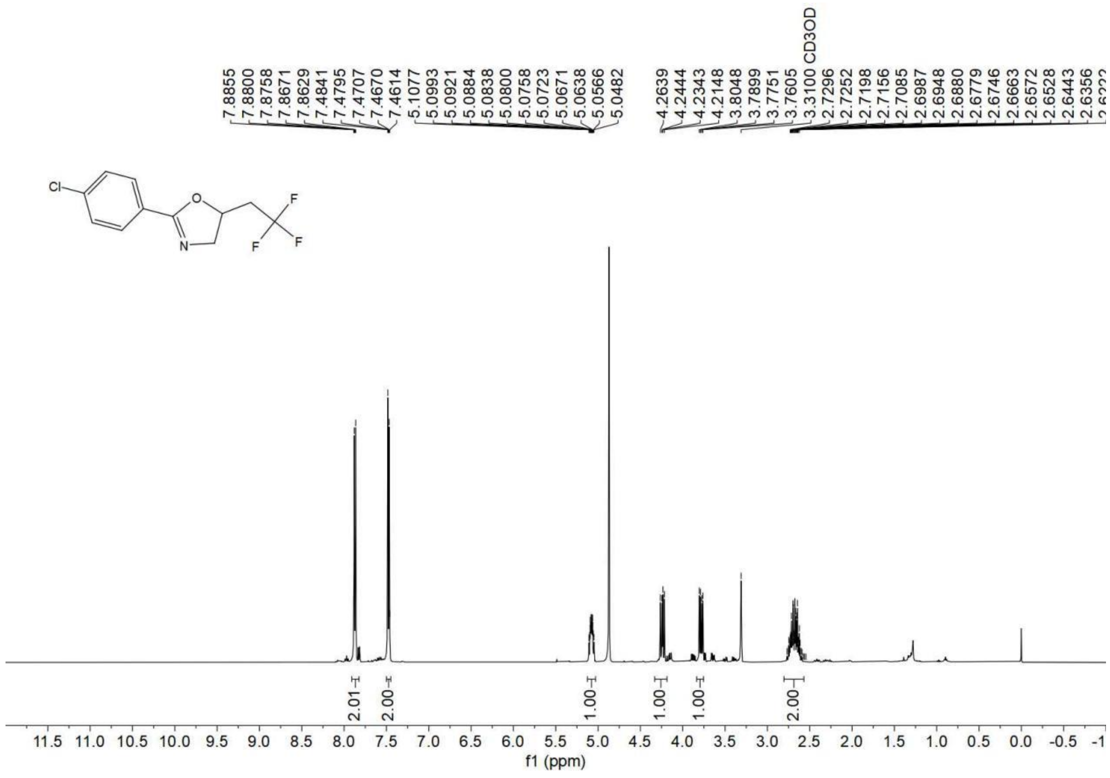

^{1}H NMR Spectrum of **4e** (500 MHz, CD_3OD)

2-(4-bromophenyl)-5-(2,2,2-trifluoroethyl)-4,5-dihydrooxazole (4f):

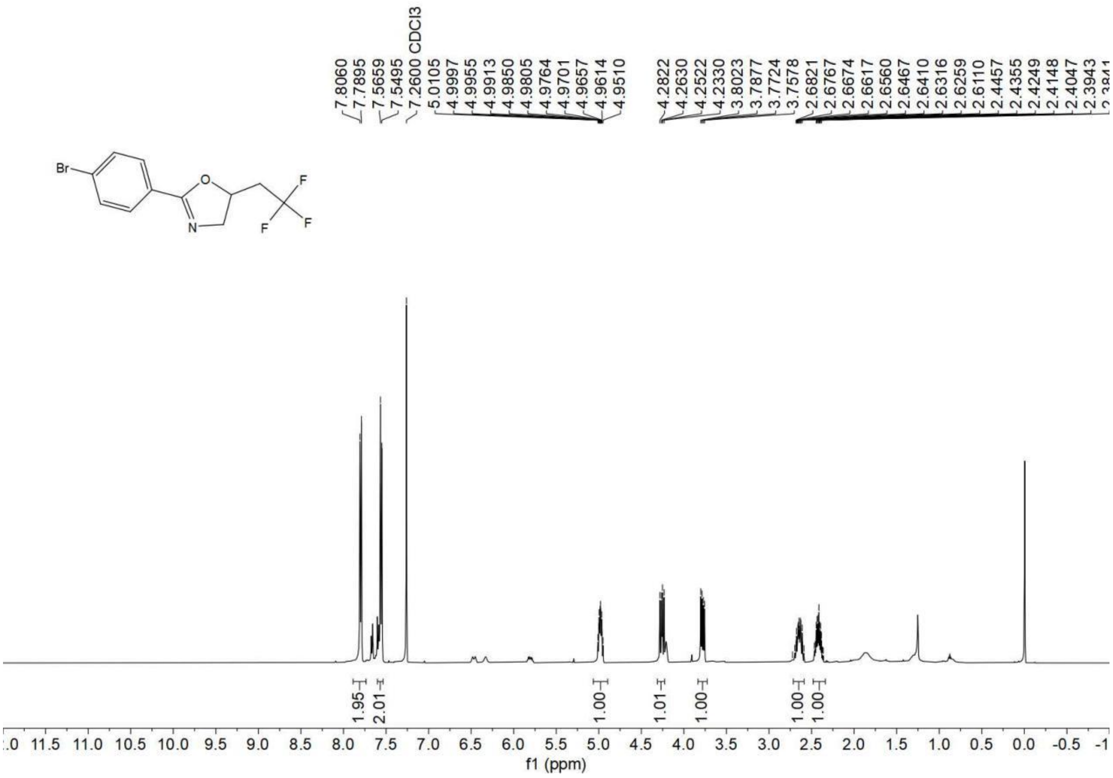

^{1}H NMR Spectrum of **4f** (500 MHz, $CDCl_3$)

2-(4-nitrophenyl)-5-(2,2,2-trifluoroethyl)-4,5-dihydrooxazole (4g):

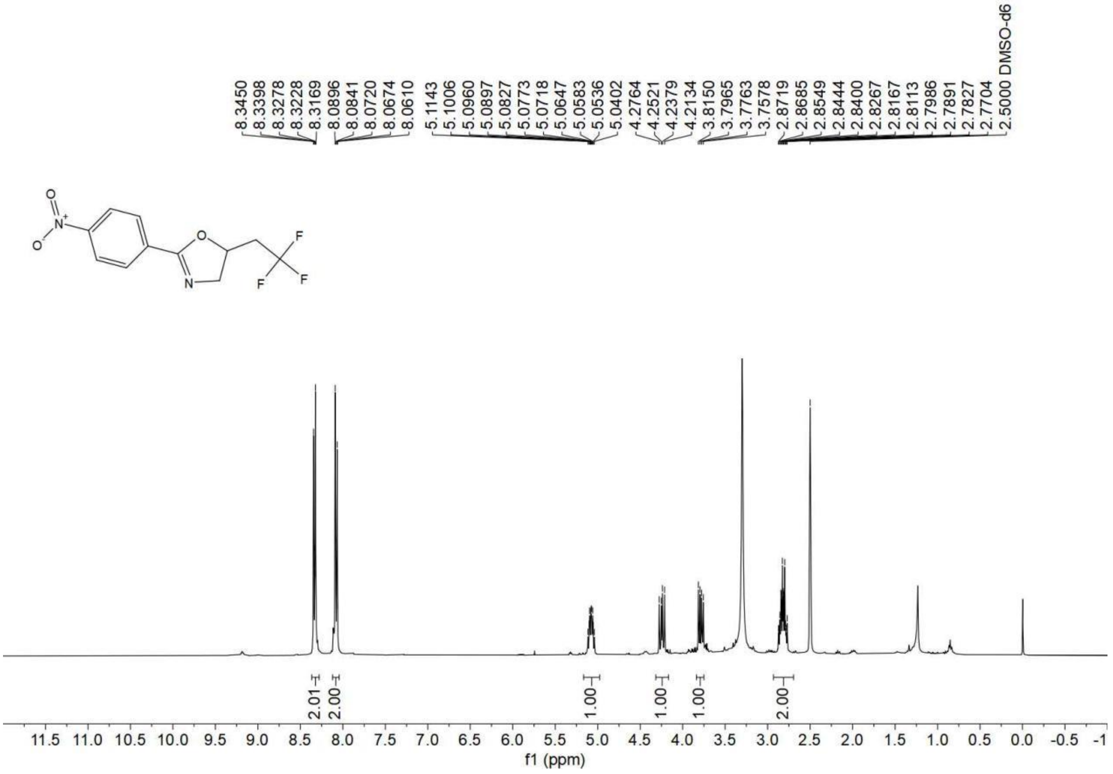

^{1}H NMR Spectrum of **4g** (400 MHz, DMSO)

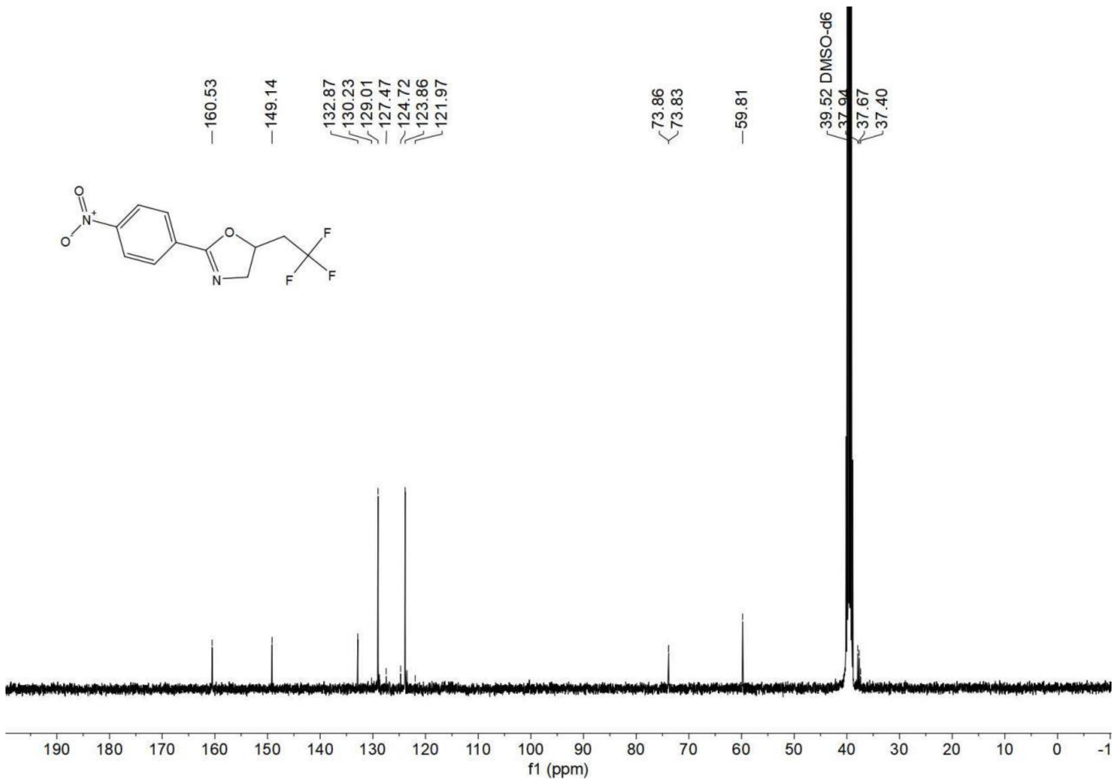

^{13}C NMR Spectrum of **4g** (100 MHz, DMSO)

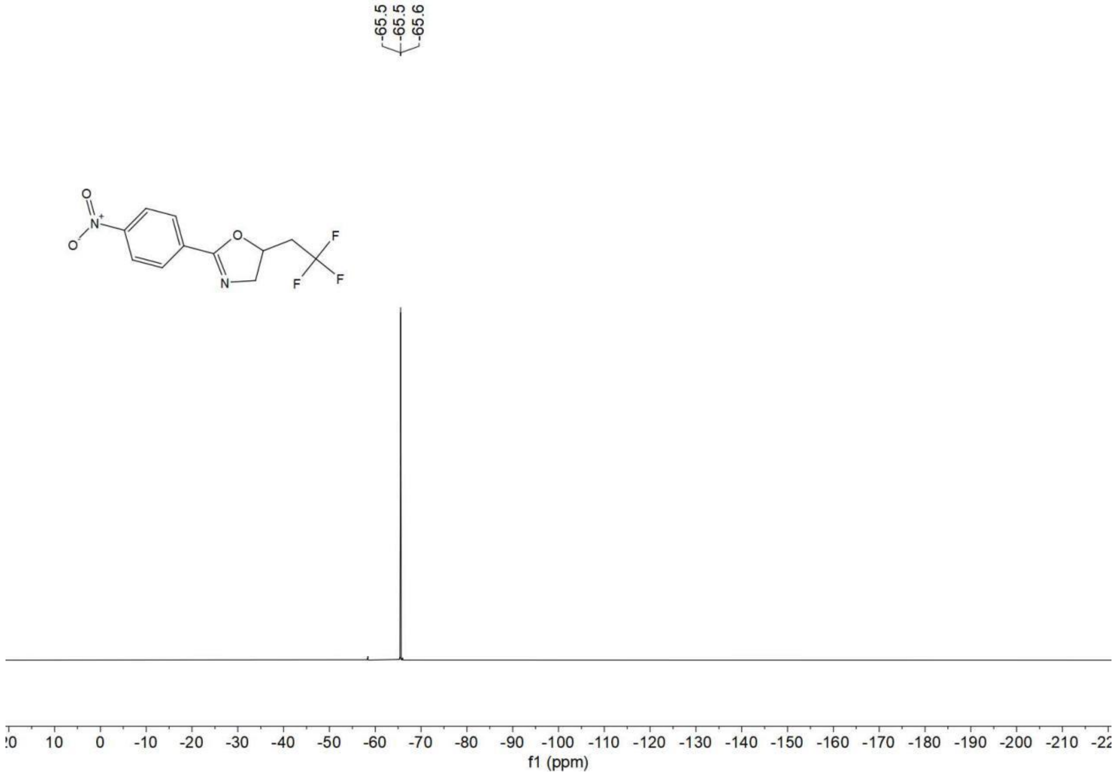

^{19}F NMR Spectrum of **4g** (470 MHz, CD_3OD)

2-(thiophen-2-yl)-5-(2,2,2-trifluoroethyl)-4,5-dihydrooxazole (4h):

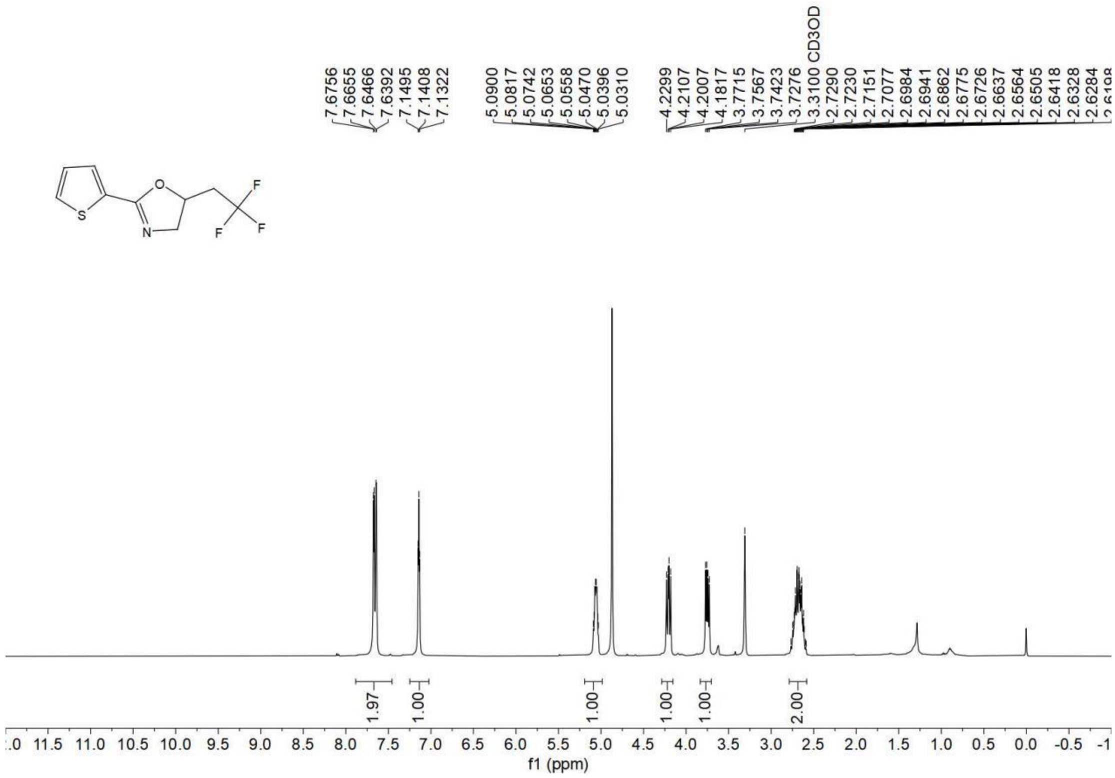

^{1}H NMR Spectrum of **4h** (500 MHz, CD_3OD)

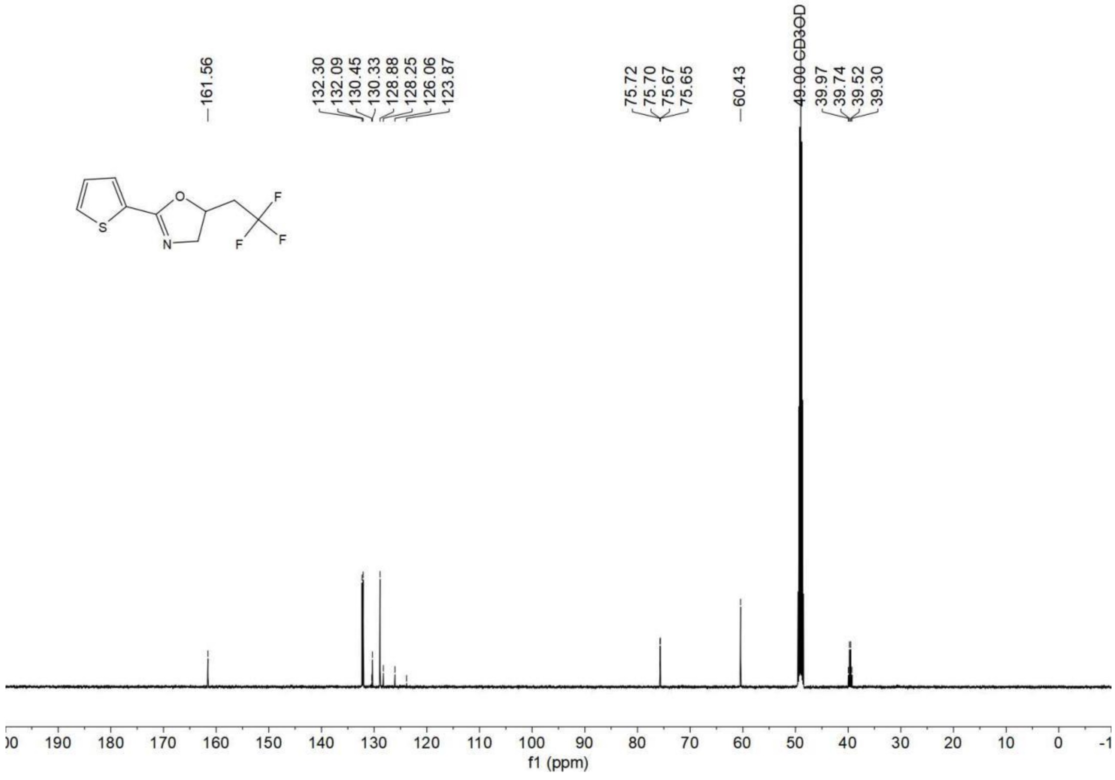

^{13}C NMR Spectrum of **4h** (125 MHz, CD_3OD)

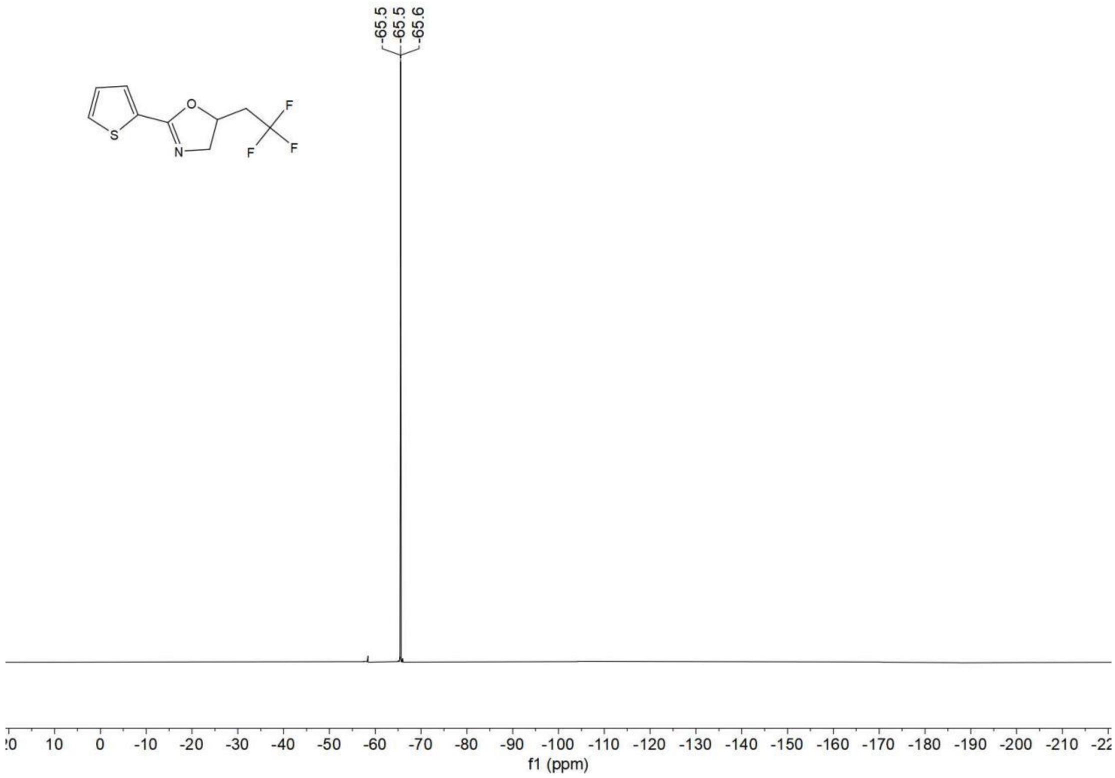

^{19}F NMR Spectrum of **4h** (470 MHz, CD_3OD)

2-(2-(benzylamino)phenyl)-4,4,4-trifluorobutan-2-ol (5):

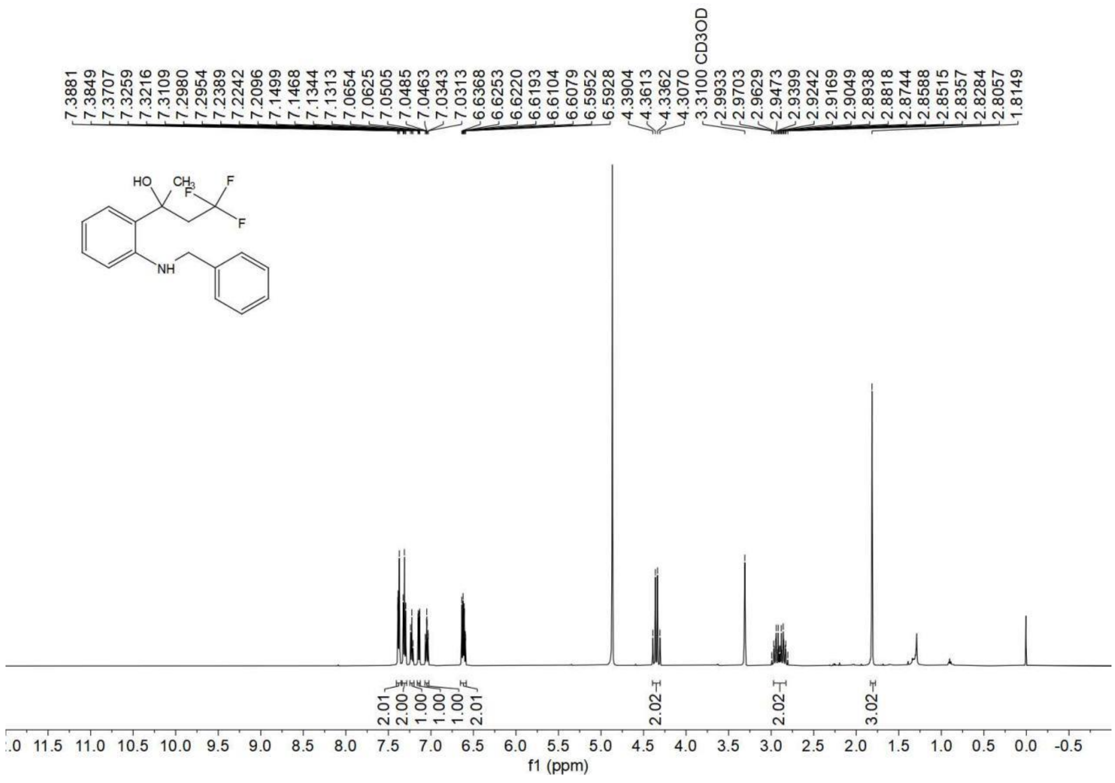

^{1}H NMR Spectrum of **5** (500 MHz, CD_3OD)

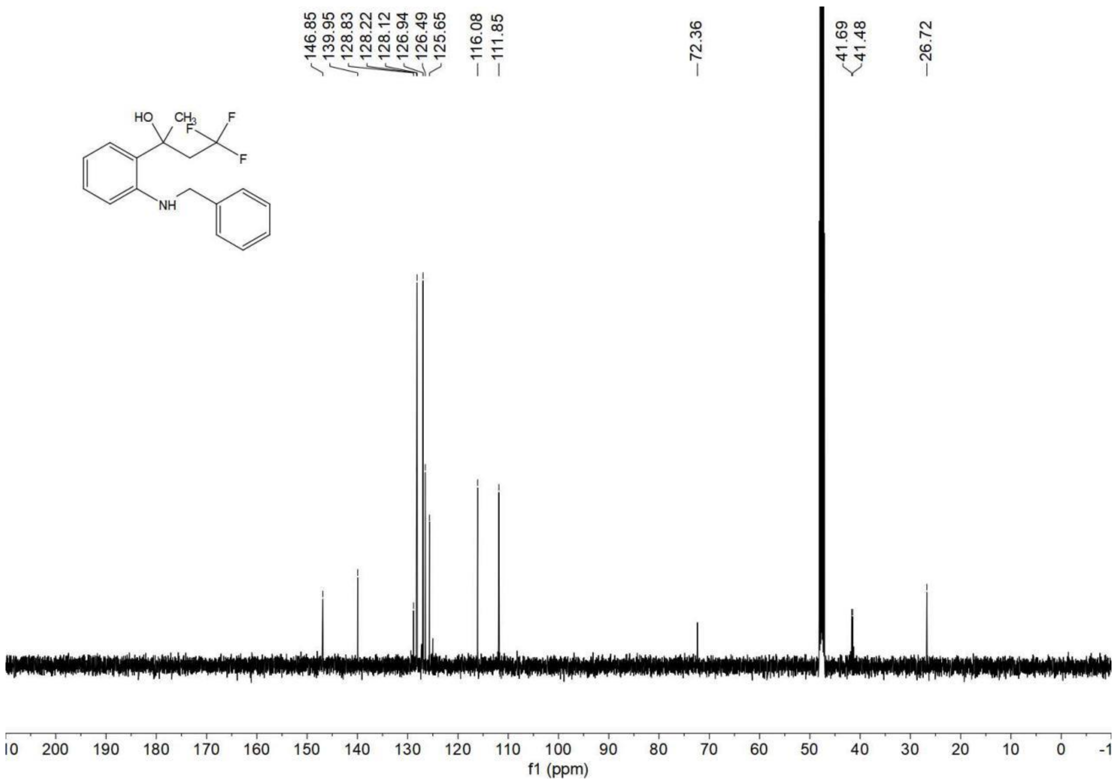

^{13}C NMR Spectrum of **5** (125 MHz, CD_3OD)

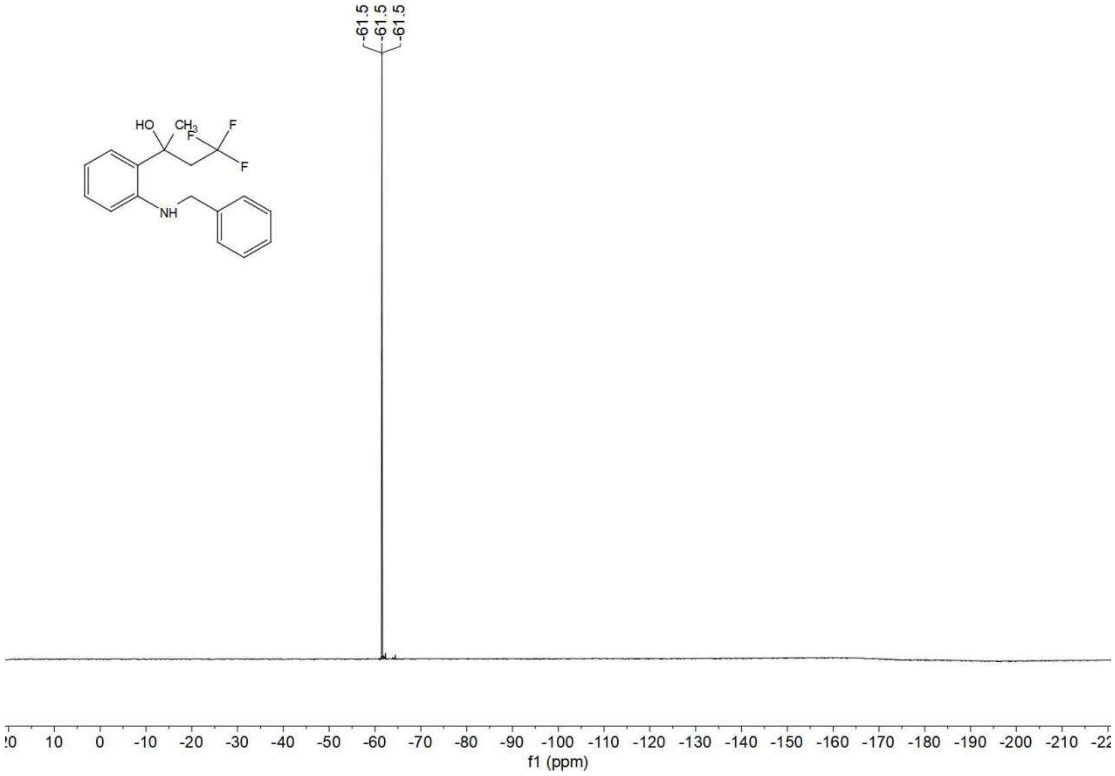

^{19}F NMR Spectrum of **5** (470 MHz, CD_3OD)

5. Cyclic Voltammogram

Cyclic voltammetry experiments were carried out in CHI1000C potentiostat. Analyte (0.02 M) or $^{n}Bu_4NPF_6$ (0.01 M) was dissolved in acetonitrile. Working electrode: glassy carbon, counter electrode: Pt, reference electrode: Ag/AgCl. Scan rate: 100 mV/s.

a) Cyclic voltammograms of $^{n}Bu_4NPF_6$ in acetonitrile (black line). b) **1a** (0.02 M), $^{n}Bu_4NPF_6$ (0.01 M) in acetonitrile (orange line). c) CF_3SO_2Na (0.02 M), $^{n}Bu_4NPF_6$ (0.01 M) in acetonitrile (green line). d) **1a** (0.02 M), CF_3SO_2Na (0.02 M), $^{n}Bu_4NPF_6$ (0.01 M) in acetonitrile (blue line).

The oxidation peak of **1a** was observed at 1.32 V. The oxidation peak of CF_3SO_2Na was observed at 0.83 V. These results indicated that CF_3SO_2Na was much easier to be electrochemically oxidized to generate fluroromethyl radicals than **1a**.

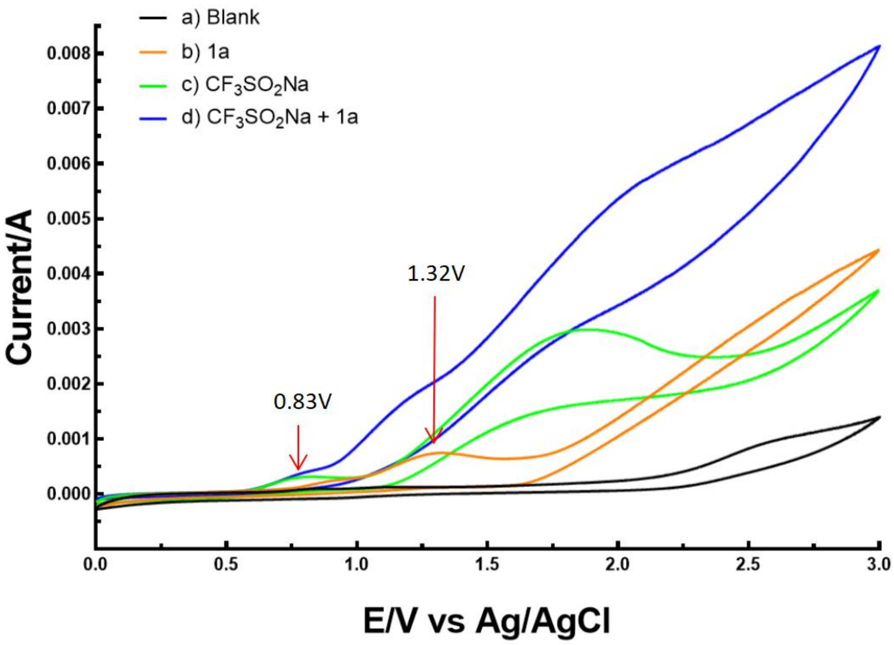

6. References

1 S. Jana, A. Ashokan, S. Kumar, A. Verma and S. Kumar, Copper-catalyzed trifluoromethylation of alkenes: synthesis of trifluoromethylated benzoxazines, *Organic & Biomolecular Chemistry*, 2015, **13**, 8411-8415.

2 S. S. Babu, A. A. Varma and P. Gopinath, Photoredox catalyzed cascade CF_3 addition/chemodivergent annulations of ortho-alkenyl aryl ureas, *Chem. Commun.*, 2022, **58**, 1990-1993.

3 S. Kawamura, D. Sekine and M. Sodeoka, Synthesis of CF_3-containing oxazolines via trifluoromethylation of allylamides with Togni reagent in the presence of alkali metal iodides, *Journal of Fluorine Chemistry*, 2017, **203**, 115-121.

4 W. Fu, X. Han, M. Zhu, C. Xu, Z. Wang, B. Ji, X.-Q. Hao and M.-P. Song, Visible-light-mediated radical oxydifluoromethylation of olefinic amides for the synthesis of CF_2H-containing heterocycles, *Chem. Commun.*, 2016, **52**, 13413-13416.
